# Hair surface interactions against different chemical functional groups as a function of environment and hair condition

**DOI:** 10.1111/ics.12834

**Published:** 2023-03-14

**Authors:** Leslie Labarre, Ophélie Squillace, Yu Liu, Peter J. Fryer, Preeti Kaur, Shane Whitaker, Jennifer M. Marsh, Zhenyu J. Zhang

**Affiliations:** ^1^ School of Chemical Engineering University of Birmingham, Edgbaston Birmingham UK; ^2^ The Procter & Gamble Company Mason Business Centre Mason Ohio USA

**Keywords:** formulation, hair treatment, polymer, spectroscopy

## Abstract

**Objective:**

The nature and magnitude of molecular interactions on hair surfaces underpin the design of formulated products, of which the application involves a competitive adsorption process between cationic surfactants, fatty alcohols and surface actives such as silicone. The knowledge of molecular interaction with hair surface will not only provide insight on the surface binding affinity but also offer an effective methodology in characterizing surface deposits.

**Methods:**

Untreated and chemically treated hair samples were treated with either conditioner chassis alone (gel network) or conditioner chassis plus silicone (chassis/TAS). Hair surface interactions against four different chemical functional groups, namely methyl (−CH_3_), acid (−COOH), amine (−NH_2_) and hydroxyl (−OH), were quantified in both ambient and aqueous environment using Chemical Force Microscopy, a method based on atomic force microscopy (AFM).

**Results:**

Surface adhesion on hair in ambient is dominated by capillary force that is determined by both the wettability of hair fibre (hydrophobic vs. hydrophilic), presence of any deposits and the chemical functionality of the AFM cantilever. Capillary force is diminished and replaced by electrostatic interaction when polar groups are present on both hair and AFM cantilever. A distinctively different force, hydrophobic interaction, plays a major role when virgin hair and hydrophobic functionalized AFM cantilever make contact in water.

**Conclusion:**

Results acquired by AFM cantilevers of different functional groups show that hydrophobic interaction is a key driver for deposition on virgin hair, whilst electrostatic interaction is the most important one for bleached hair. Interfacial conformation of chassis components upon deposition is determined by the hair surface properties. Our study highlights the possibility of a range of polar groups, not necessarily negatively charged, on the damaged hair. Unlike conventional surface chemical analysis method, it is possible to quantitatively evaluate the interfacial conformation of deposited surface actives on hair, which identifies the target moieties for conditioning products on different types of hair.

## Résumé

**Objectif:**

La nature et l'intensité des interactions moléculaires mesurables à la surface d'un cheveu, caractérisent l'effet de la formulation du produit initialement appliqué sur le cheveu. L'application du produit et son effet sur le cheveu repose sur un mécanisme d'adsorption complexe combinant l'effet de différents éléments tels que des surfactants cationiques, des alcools gras et des agents de surface tel que le silicone. L'étude et l'analyse des interactions moléculaires à la surface du cheveu permettent non seulement de déterminer l'affinité adhésive de surface, mais aussi d'offrir une méthode efficace pour caractériser les dépôts de surface.

**Méthode:**

Des cheveux initialement traités et non‐traités chimiquement, ont été analysés après l'application d'un après‐shampoing chassis (structure en gel) ou d'un après‐shampoing chassis avec du silicone (chassis/TAS). Les interactions entre la surface du cheveu et quatre groupes fonctionnels chimiques – méthyle (–CH_3_), acide (–COOH), amine (–NH2) et hydroxy (–OH) ont été quantifiées à l'air et en milieu aqueux par microscopie à force chimique, une méthode basée sur la microscopie à force atomique (AFM).

**Résultats:**

L'adhésion de surface sur cheveu à l'air est dominée par la force capillaire qui est déterminée par la mouillabilité de la fibre capillaire (hydrophobe vs. hydrophile), la présence de dépôts, et la fonction chimique du cantilever. La force capillaire diminue et est remplacée par des interactions électrostatiques quand des groupes polaires sont présents à la fois sur le cheveu et le cantilever. Une autre force – l'interaction hydrophobe, joue un rôle majeur quand un cheveu non‐traité / vierge et un cantilever de fonction hydrophobe se rencontrent en milieu aqueux.

**Conclusions:**

Les résultats obtenus à partir de cantilevers de différentes fonctions chimiques, montrent que l'interaction hydrophobe joue un rôle clé dans l'application de produit capillaire sur cheveux non‐traité tandis que les interactions électrostatiques sont prédominantes dans le cas de cheveux traités chimiquement. La conformation interfaciale des composés chassis avant déposition est déterminée par les propriétés de surface du cheveu. Notre étude souligne la présence potentielle de différents groupes polaires, pas nécessairement chargés négativement, sur la surface de cheveux endommagés. A l'inverse des méthodes conventionnelles d'analyse chimique de surface, il est possible d'évaluer quantitativement la conformation interfaciale de dépôts d'agents actifs présents à la surface du cheveu, ce qui permet d'identifier les zones cibles pour l'application d'après‐shampoing sur différents types de cheveux.

## INTRODUCTION

Surface quality of hair is critical to the design of hair care products: untreated (virgin) hair is relatively hydrophobic due to the presence of a compact layer of lipids (18‐methylicosanoic acid) on the surface [1, 2], but becomes more polar (and hydrophilic) once the lipid layer is damaged or removed (partly or completely) via per‐hydrolysis upon treatment of oxidative colourants and bleaches [3]. The altered surface properties result in hair fibres that are rough and readily tangled, which could be addressed by applying conditioning products. A common design principle for hair conditioner formulation is to deposit surface actives via a gel network that is called chassis (consisting of cationic surfactants, fatty alcohol and water) on to hair fibres [4, 5]. As a competitive surface adsorption process between a range of compounds, successful deposition of conditioning actives is determined by their molecular interactions with hair, which is controlled by the chemical functional groups available on hair fibres, molecular architecture of the active compounds, conditioner formulation, etc.

Previous studies suggest that there are negatively charged groups present on damaged hair [3], and hence molecules containing positively charged moieties are commonly used as surface actives in haircare products [6]. The difference between damaged and undamaged regions on the same hair results in a considerable technical challenge: what chemistry could work well on both untreated hair, and also on chemically treated hair [6]. As suggested in the literature, one strategy to compensate for the poor affinity of hydrophobic actives such as dimethicones on damaged hair is to use cationic polymeric agents [6]. However, such deposition process is a complex colloidal phenomenon that requires cationic conditioning agents being included in the formulated products in the presence of oppositely charged anionic surfactants [7]. All the technical solutions so far highlight the importance of understanding the molecular interactions between functional actives and hair fibres of varied treatment.

Conventional surface analysis techniques, such as secondary ion mass spectroscopy [8], X‐ray photoelectron spectroscopy [9] and infrared spectroscopy [10, 11], are excellent in quantifying the presence of chemicals of interest on hair surface in both vacuum and ambient environment. These are complemented by techniques such as interfacial tensiometry and scanning electron microscopy that measure physical characteristics, for example, surface free energy and morphology respectively [12, 13], of hair fibres prior to and post‐treatment. However, designing an effective surface active for chemically treated hair remains a challenge due to the mismatched capabilities between the aforementioned techniques: there is a limited knowledge concerning the orientation of the molecules deposited on the hair surface and the molecular arrangement of the adsorbed layer on real hair, which could be addressed by atomic force microscopy (AFM) [14, 15, 16, 17].

With the ability to work on complex objects, including hair, in both ambient and liquid environments [18], at nanometre spatial resolution [19], AFM had been used to investigate the surface morphology of hair fibres for the past two decades [20]. It is possible to generate correlating information of surface morphology and mechanical properties of hair surface simultaneously with the recent advances made in AFM technique, such as quantitative nanomechanical imaging [21, 22, 23, 24]. A unique feature of AFM is its capability of quantifying surface interactions [25] with piconewton sensitivity. When two surfaces are in contact, the surface force consists of multiple components [26, 27], including van der Waals (VDW) forces, hydrogen bonding, electrostatic interactions, steric repulsion, hydrophobic interactions and capillary force, the nature and magnitude of which is dependent on the environment where contact is being made. For example, electrostatic interaction plays a dominant role in a colloidal system where objects are charged, depending on their chemical nature. For surface adhesion in ambient, the most dominating factor is capillary force and/or viscous force, where the presence of a layer of condensed water or a viscous fluid increases the energy required to separate the two surfaces in contact [28]. AFM studies have been carried out to evaluate the nanomechanical properties, including surface adhesion, modulus and lubrication, of hair fibres, which provides invaluable insights relating chemical treatment and surface characteristics of hair [9, 20, 29, 30]. In some studies, a small piece of hair fibre was attached to the end of an AFM cantilever, so that fibre–fibre interactions could be directly quantified [31], which demonstrates the flexibility of the AFM technique. Most of the previous work has been focused on the characterization of hair, with or without treatment. We believe that such AFM‐based approach could be instrumental to the design and selection of surface actives because the molecular interaction on fibre surface underpins deposition and resulting interfacial conformation of the actives [24, 32].

In the present work, two types of hair, virgin hair (VH) and platinum‐bleached hair (PTB), were selected as representative systems: VH is a hair sample with no previous chemical treatment as representative of consumers who do not colour or bleach their hair, and PTB is a sample that had undergone oxidative damage using a hydrogen peroxide/ammonium persulfate bleach as representative of consumers who frequently colour or bleach their hair. Both VH and PTB were treated with either chassis alone (gel network) or conditioner chassis plus 2% of a terminal amino‐silicone (chassis/TAS). TAS is a linear dimethicone that is capped at both ends with a propylamine group; viscosity of ~10 000 cps. We quantitatively measured the interactions between the hair fibres (VH vs. PTB) and chemical functional groups that are relevant to the current conditioning actives in both ambient (air) and deionized water (liquid). Four different thiol‐based self‐assembled monolayers (SAMs) were prepared on gold‐coated AFM cantilevers [33]: (i) methyl (−CH_3_) monolayer to render a hydrophobic coating; (ii) carboxylic acid (−COOH) monolayer as a hydrophilic coating; (iii) hydroxyl (−OH) monolayer to replicate fatty alcohol; and (iv) amine (−NH_2_) monolayer to replicate amino‐silicone. A combination of such surface chemistry can help differentiating the factors that contribute towards hair surface interactions, which underpins the development of new, sustainable, eco‐friendly hair care products.

## MATERIALS AND METHODS

### Materials

Chemically untreated Caucasian‐source virgin hair and platinum‐bleached hair were purchased from International Hair Importers & Products Inc. (Glendale, NY). Platinum‐bleached hair was produced from dark brown Caucasian hair by first soaking hair in a solution of ammonium hydroxide/hydrogen peroxide at pH 10 for 90 min until the cysteic acid levels were similar to the values from consumers who use powder bleach products, followed by treatment with an ammonium persulfate/hydrogen peroxide powder bleach for 20 min. The hair samples were rinsed thoroughly after exposure to the oxidant (>20 min) to remove any residual alkalinity from the bleaching procedure. Individual tresses (4 gm, 8 in with hot wax tab at top), formed by evenly blending hair from multiple ponytails, were used for all experiments.

Six samples, including VH and PTB, treated by a conditioner chassis containing cetyl alcohol (4%), stearyl alcohol (3%) and stearamidopropyl dimethylamine (SAPDMA) quat (3%), and the same conditioner chassis with the addition of 2% amodimethicone 10 000 cps (TAS), were examined. Each sample contains three tress replicates. Each treatment consisted of three wash cycles where each cycle was application of a non‐conditioning clarifying shampoo (0.1 g shampoo per g of hair) that was milked into hair for 30 s, rinsed for 30 s, followed by the conditioner (0.1 g conditioner per g hair) that was milked for 30 s and rinsed for 30 s. The hair was dried in a hot box where 80°C air is circulated to mimic blow drying between each wash cycle.

For AFM cantilever functionalization, 11‐mercapto‐1‐undecanol (>99%), dodecanethiol (>98%), 11‐amino‐1‐undecanethiol hydrochloride (>99%), 12‐mercaptododecanoic acid (>96%), trifluroacetic acid (>99%), triethylamine (>99%), hydrochloric acid (reagent grade, 37%) and absolute ethanol (99%) were purchased from Sigma‐Aldrich and used as received.

### Atomic force microscopy

An atomic force microscope (Multimode, Veeco, UK) with a Nanoscope IV controller was used for characterizing the hair samples in both ambient and liquid. Gold‐coated cantilevers with a nominal spring constant of 0.32 N m^−1^ (PNP‐TR‐Au, Nanoworld, Switzerland) were used to acquire surface morphology and surface interactions. Force curves were collected over different regions with a 2 nN applied load.

During a force measurement, AFM cantilever is positioned above the hair sample that is fixed to a piece of indium, as described in a previous work [34]. It subsequently approaches the hair fibre in the normal direction, makes contact before retracting from the hair surface at a defined frequency. Surface interactions between the cantilever and the hair fibre are registered during this process via the movement of laser beam that is focus on the backside of the cantilever onto a photodetector. A detailed explanation can be found in a review article by Butt and colleague [27]. By treating the cantilever as a spring, it is possible to translate the electronic signal recorded by the photodetector to an actual force, with rigorously calibrated deflection sensitivity and spring constant of the cantilever. Results generated are usually presented as force recorded as a function of the z piezo displacement that is the relative distance between the cantilever tip and the hair fibre.

### AFM cantilever functionalization

All cantilevers used were cleaned by submersion in piranha solution, a mixture of hydrogen peroxide and concentrated (95%) sulphuric acid at a 3:7 ratio (***Caution!*** *Piranha solution is an extremely strong oxidizing agent that has been known to detonate spontaneously upon contact with organic material*.). Subsequently, the cantilevers were rinsed thoroughly with deionized water and dried in an oven. The cleaned gold‐coated cantilevers were placed in a clean petri dish containing the prepared thiol solutions for a minimum of 18 h to ensure a complete formation of the monolayer.

To prepare self‐assembled monolayer of methyl (−CH_3_) or hydroxyl (−OH) functionality, dodecanethiol or 11‐mercapto‐1‐undecanol were dissolved in degassed ethanol at a concentration of 1 mM respectively. To prepare acid (−COOH) SAMs, 1 mM of 12‐mercapto‐dodecanoic acid was prepared using a 2% v/v trifluroacetic acid and ethanol mixture [33]. After 24 h immersion, the cantilevers were rinsed by ethanol to remove excess solution, followed by a 10% v/v ammonia in ethanol solution to remove any residual trifluroacetic acid, and finally copious amounts of ethanol before storage. For amine SAM, a mixture of 0.1 mM 11‐amino‐1‐undecanethiol hydrochloride and 1 mM triethylamine in ethanol was used.

### Surface analysis

Deposition of conditioning materials on hair was measured by extraction followed by quantification. For all samples, 0.1 g of hair was sampled for extraction with three locations per tress (nine measurements in total). Silicone was extracted using a mixture of 50:50 methylisobutylketone:toluene and analysed by ICP‐OES (Optima 5300 DV, Perkin Elmer, Shelton, CT, USA). Fatty alcohol was extracted with a mixture of 50:50 chloroform: methanol and analysed by GC‐FID with a flame ion detector (Agilent 6890, CA, USA). SAPDMA was extracted with a mixture of 20:20:60 methylisobutylketone:methanol:hexane and analysed by LC‐CAD (Agilent 1260 with Diode Array Detector, CA, USA).

### Surface energy

Surface energy measurements were conducted using a Krüss Tensiometer K100 SF (Krüss, Hamburg, Germany) under environmental conditions of 23 ± 1°C and 50 ± 4% RH. The method developed by Franz J. Wortmann and colleague was deployed to determine advancing water contact angles on hair fibres [12]. A small segment of hair (middle region of a fibre) is mounted to a microbalance before being perpendicularly immersed into a solvent of known properties (density, surface tension and viscosity). The mass of solvent interacting with the hair segment, quantified by the microbalance, is proportional to the wetting force by the gravitational constant. Wilhelmy Equation was used to convert the wetting force measured into contact angles that were subsequently expressed as surface energy using the Owens–Wendt (O‐W) method.

## RESULTS AND DISCUSSION

### Surface deposition analysis

Virgin and PB hair fibres with or without treatment, including both chassis only and chassis/TAS, were quantitatively examined by surface chemical analysis. The presence of silicone and cationic surfactant (SAPDMA) was quantified in μg g^−1^, as shown in Table 1. It can be seen that there was a trace amount of silicone on both virgin and PB hair, which changed slightly upon treatment by chassis alone. The deposition amount of silicone on chassis/TAS treated hair samples vary significantly: a trace amount of silicone was observed on PTB hair, which contrasts distinctively to the notable amount observed on the VH sample.

**TABLE 1:** Silicone and cationic surfactant deposition in μg g^−1^ as a function of hair type (virgin hair, platinum‐bleached hair) and treatment (chassis alone, chassis/TAS) in ambient air. Three hair fibre samples were collected from three different hair tresses, resulting in a total of nine measurements for each type.

	Hair type	Untreated hair	Chassis alone	Chassis / 2% TAS
Silicone	VH	26 ± 5	71 ± 9	1273 ± 676
Cationic surfactant (SAPDMA)	VH	0	252 ± 31	150 ± 33
Total Fatty Alcohol	VH	339 ± 20	1298 ± 143	616 ± 77
Silicone	PTB	24 ± 16	10 ± 1	74 ± 21
Cationic surfactant (SAPDMA)	PTB	0	214 ± 32	258 ± 41
Total Fatty Alcohol	PTB	188 ± 12	817 ± 108	398 ± 130

### Hair surface free energy

Measurements were made using water and hexadecane to calculate both polar and non‐polar components of the hair surface free energy. Although the values of non‐polar component are very close for both VH and PTB hair samples, the latter has a substantial polar component (42.1 mJ m^−2^), which confirms that VH sample is hydrophobic and PTB sample is hydrophilic. This observation is consistent with those reported in previous studies [6].

### Surface morphology of hair fibres

**FIGURE 1:**
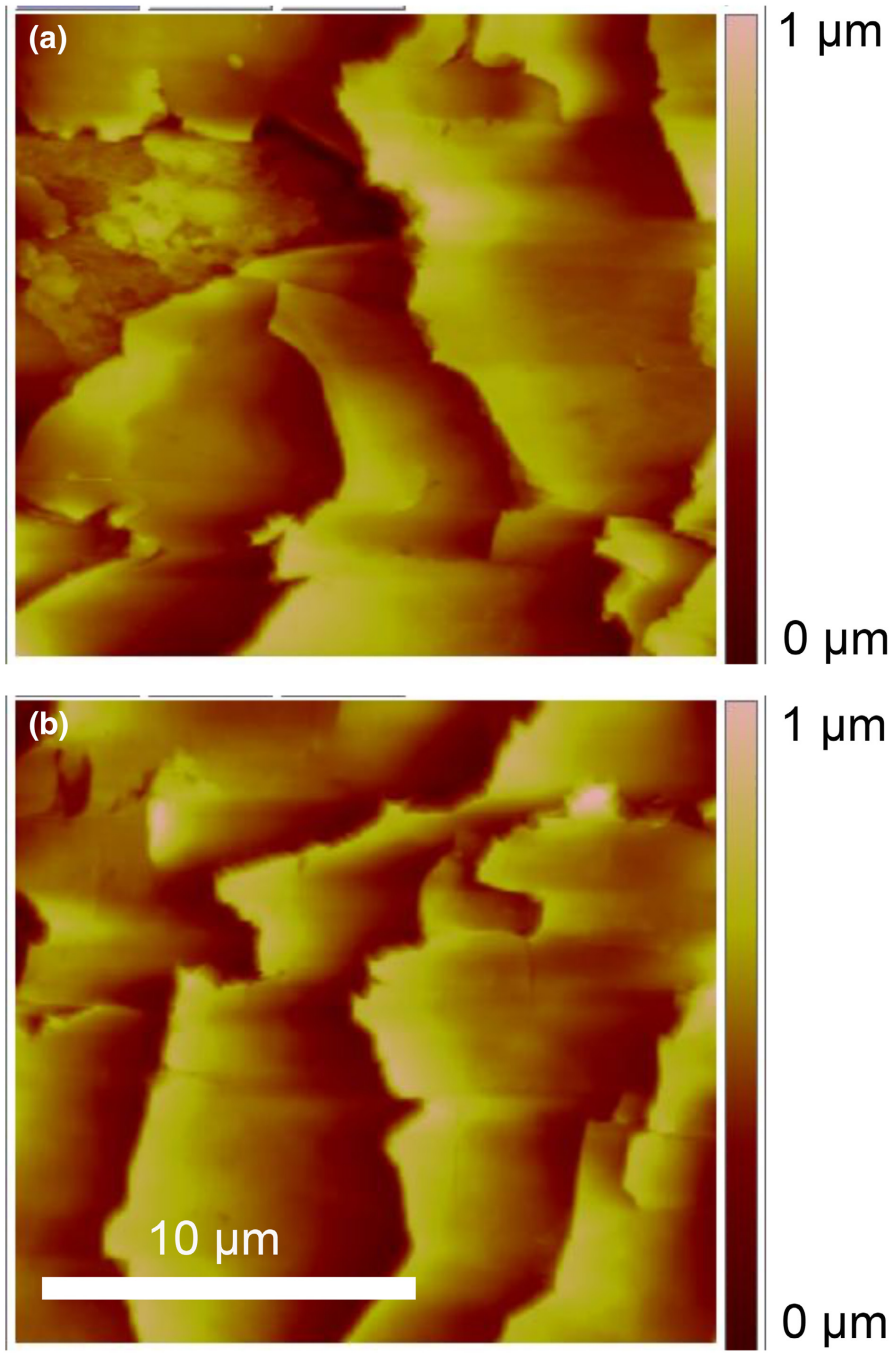
AFM images of middle region of untreated hair acquired in (a) ambient and (b) water.

To establish the surface morphology of hair fibres provided, AFM was used with a scan size of 20 μm by 20 μm, of which representative images are shown in Figure 1. At least five locations on different fibres were surveyed for each sample to ensure the surface features observed are representative and reproducible. To minimize any potential inconsistency between hair samples, all measurements were carried out in the middle region. This protocol evaluates the integrity of the fibre surface, and makes sure that the subsequent measurements can be acquired over reliable locations. Similar to a previous work concerning the physical appearance of hair fibres, repetitive cuticles are shown in the images (both ambient and water). The results provide assurance that the AFM cantilever was in regular contact with the hair fibre and that experimental protocol was robust.

Subsequently, surface adhesion was quantitatively measured by the AFM‐based force spectroscopy using functionalized tip of different chemistry. There were six samples, including two types of hair, VH and PTB, with and without chassis and chassis/silicone treatment, which were examined in two different media: air and water.

### Hair surface adhesion against methyl (−CH_3_ ) functional group

**FIGURE 2:**
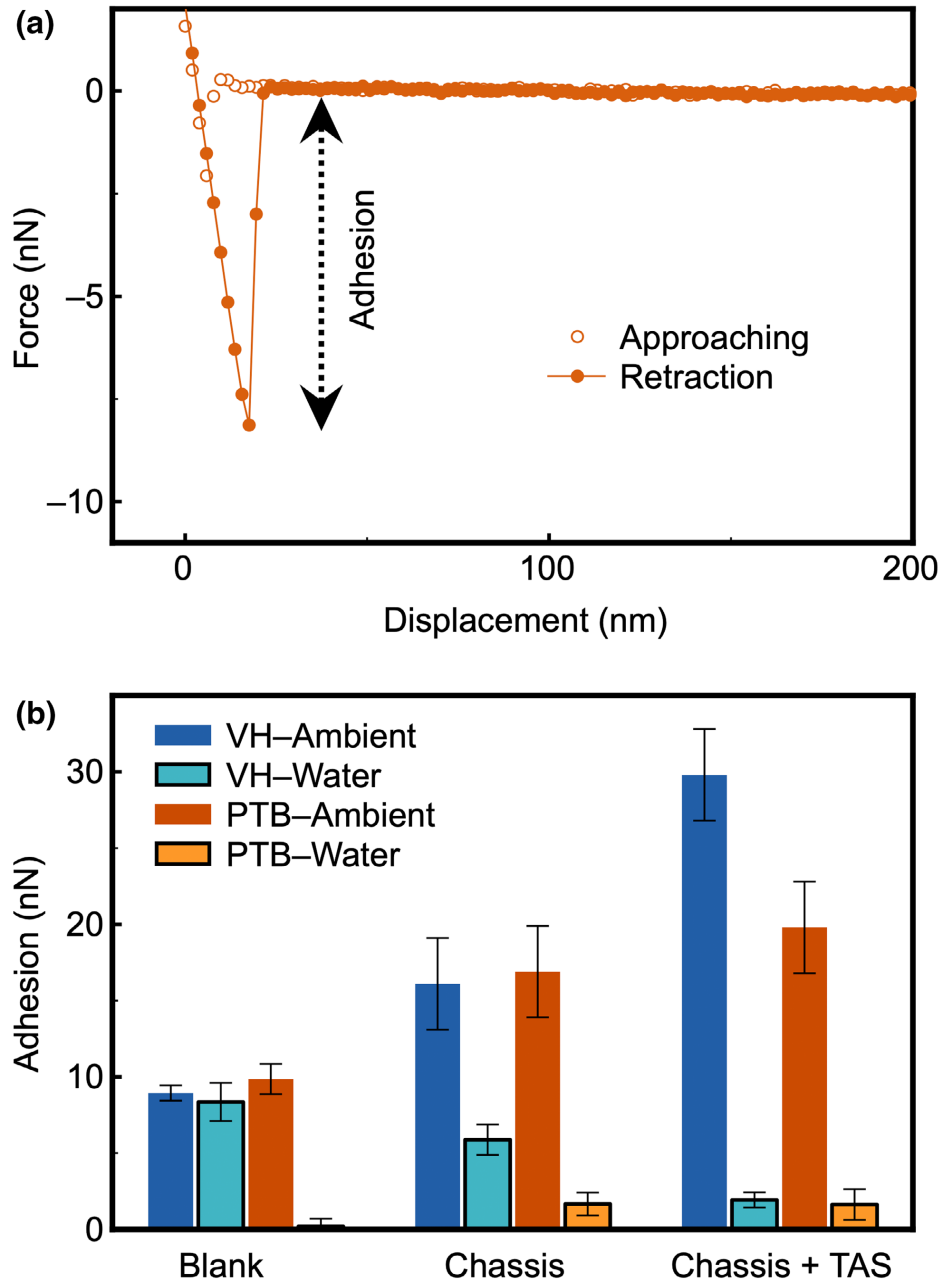
(a) Representative force curves acquired using a methyl (−CH_3_) functionalized cantilever on a virgin hair (VH) in ambient. (b) averaged surface adhesion collected on all six types of hair samples in both ambient and water, using the methyl functionalized cantilevers.

**FIGURE 3:**
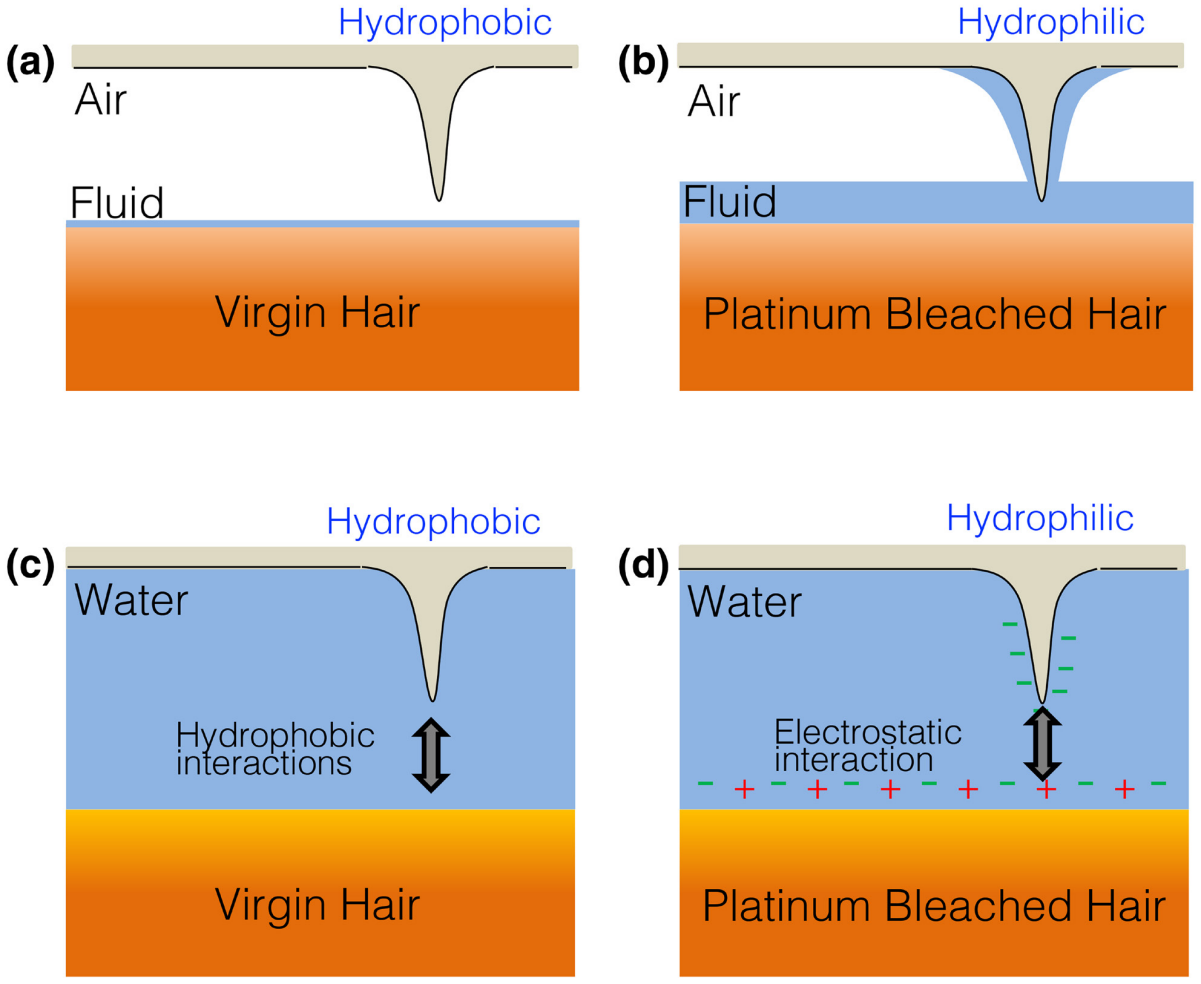
Schematic diagrams demonstrating (a) capillary force on virgin hair using a hydrophobic cantilever; (b) capillary force on PTB hair using a hydrophilic cantilever; (c) hydrophobic interaction between two hydrophobic surfaces (cantilever and hair) in water and (d) electrostatic interaction between charged surfaces in water.

As the first set of surface interaction experiments, −CH_3_ terminated AFM cantilevers were deployed to quantify the surface interaction on all six‐hair samples (middle region). A representative force curve is shown in Figure 2a, whilst the averaged surface adhesion collected by the −CH_3_ terminated AFM tips are presented in Figure 2b. When being measured in ambient, PTB explicitly shows a great adhesion towards the −CH_3_ cantilever, which is consistent with the surface free energy data that confirms the PTB is hydrophilic [8, 35]. This is because capillary force dominates the surface interaction in ambient environment [27, 34], which can be used to evaluate quantitatively the hydrophobicity of the hair samples surveyed. As demonstrated in Figure 3, a hydrophilic surface is expected to have more condensed water on its surface and therefore a stronger adhesion force result from the wetting of the tip by capillarity, which has been demonstrated in a previous work [25].

Upon the deposition of chassis, both VH and PTB hair samples result in an increased surface adhesion in ambient condition, suggesting that the hair surface becomes more hydrophilic. This is highly likely due to the presence of surfactant and fatty alcohol that are polar and more readily to attract water molecules from the ambient, forming a molecularly thin capillary layer.

The dominating mechanism of capillary force with overall surface interaction is equally applicable when the water film is replaced by a viscous fluid, such as silicone oil, as shown in Figure 3b [28]. The magnitude of the viscous force is determined by the viscosity of the fluid, as well the thickness of the fluid film, and radius of the contact. For hair samples treated with a complete conditioning formulation (chassis/TAS) in ambient, a notable increase in the surface adhesion was observed on VH, which is highly likely due to the presence of silicone that introduces viscous force rather than capillary force. The introduction of silicone did not seem to result in much difference on PTB hair samples, which is consistent with the deposition data (Table 1) that suggests a much greater amount of Si on VH than on PTB.

Force measurements on hair samples were subsequently performed in de‐ionized water, which eliminates the contribution of capillary force towards the overall surface adhesion. As the result, two other forces, hydrophobic interaction and electrostatic interaction, were induced, which would influence the overall adhesion, as illustrated in Figure 3c,d. For the cantilever functionalized with methyl (−CH_3_) groups, it is hydrophobic and thus has no polar groups to engage with the electrostatic interaction. It was found that the surface adhesion on VH in water remains the same magnitude to that in ambient, despite that capillary force no longer exists. This is due to the contribution of hydrophobic interaction, which solely takes place between two hydrophobic surfaces in aqueous environment [36]. On PTB, a negligible surface interaction was observed in water, which is due to the absence of all possible surface interactions except VDW forces.

Unlike that in ambient, treatment of chassis results in a decreased surface adhesion on VH hair sample. This is not surprising because the deposited chassis molecules are charged, consequently would reduce the magnitude of hydrophobicity of VH, and hence decrease the overall adhesion. The surface adhesion on VH in water reduces even further upon treatment of chassis/TAS, suggesting that amino‐silicone was able to (i) significantly alter the hydrophobicity of VH; (ii) block the hydrophobic interaction observed on the untreated VH sample, and (iii) expose polar groups to the water environment. A minimal adhesion was observed on PTB samples treated by chassis or chassis/silicone, when the PTB hair samples were immersed in water. It evidences that there is very little amount of silicone present on the PTB surface.

**FIGURE 4:**
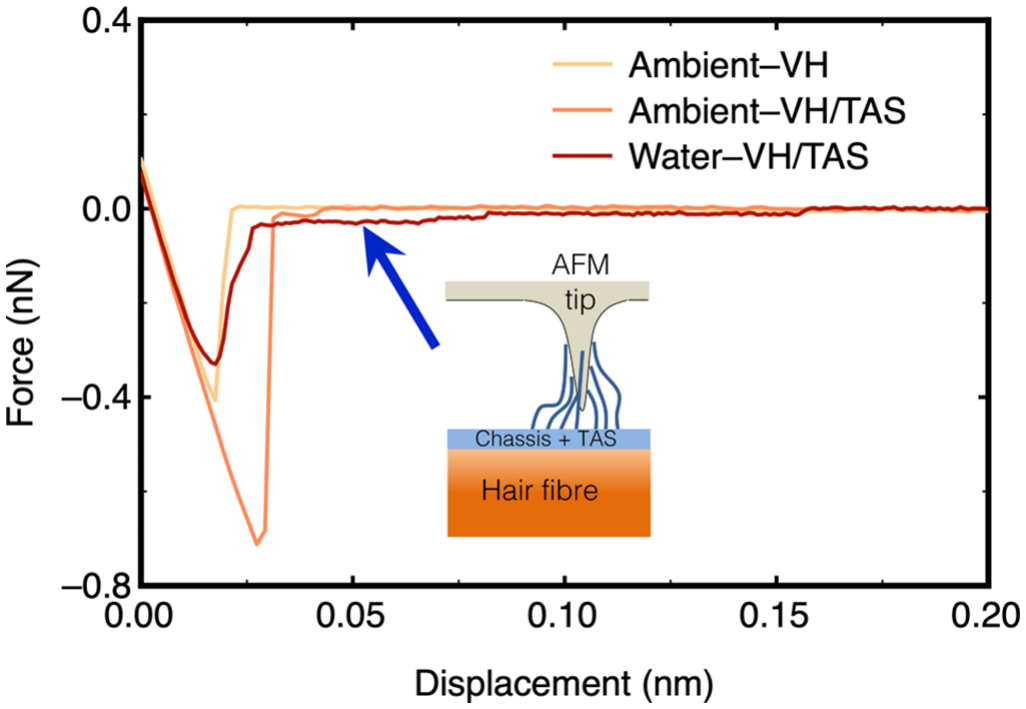
Representative retraction curves acquired from VH in ambient, and VH treated by chassis/silicone in ambient and water. The prolonged force signal following the peak (indicated by the blue arrow) suggests that there is a stretching of silicone chains, as illustrated by the inset.

Additional information concerning molecular arrangement on hair samples could be extracted from the force curves. The retraction part of the force curves on VH in ambient, and those treated by chassis/TAS in ambient and water show distinctive characteristics (Figure 4). It is worth noting that the retraction curve does not return to baseline once the tip overcomes the surface adhesion on chassis/TAS treated hair, which suggests that the contact between AFM cantilever and hair was not a brief thermodynamic process that overcomes the adhesive energy but stretches the surface deposited silicone chains as it withdraws. This not only confirms the presence of TAS on hair surface, but also supports that there could be more TAS on VH than on PTB, or that the deposited TAS adapts a less rigid molecular configuration on VH than that on PTB.

A rather counterintuitive observation is that the surface adhesion values in water are close on both PTB and VH treated with full conditioner formulation, which is inconsistent with the adhesion data acquired in ambient (Figure 2b). There are two possibilities: (i) the deposited silicone molecules could entangle with each other, driven by hydrophobic interactions between themselves, leading to either an inhomogeneous distribution on the negatively charged sites of hair, or a compact film with the amino groups buried in the molecular aggregates; (ii) the minimal adhesion between TAS and −CH_3_ in water makes it impossible to differentiate the thick and thin silicone film formed on the hair surface.

### Hair surface adhesion against carboxylic acid (−COOH) functional group

To evaluate the effect of capillary force and identify precise contributions from each component of the overall adhesion, carboxylic acid (−COOH) functionalized cantilevers were used. Comparing to the methyl (−CH_3_) functional groups, the acidic functionalization would enhance capillary force (more hydrophilic than −CH_3_) in ambient, introduce electrostatic interaction when being immersed in water, and eliminate hydrophobic interactions.

**FIGURE 5:**
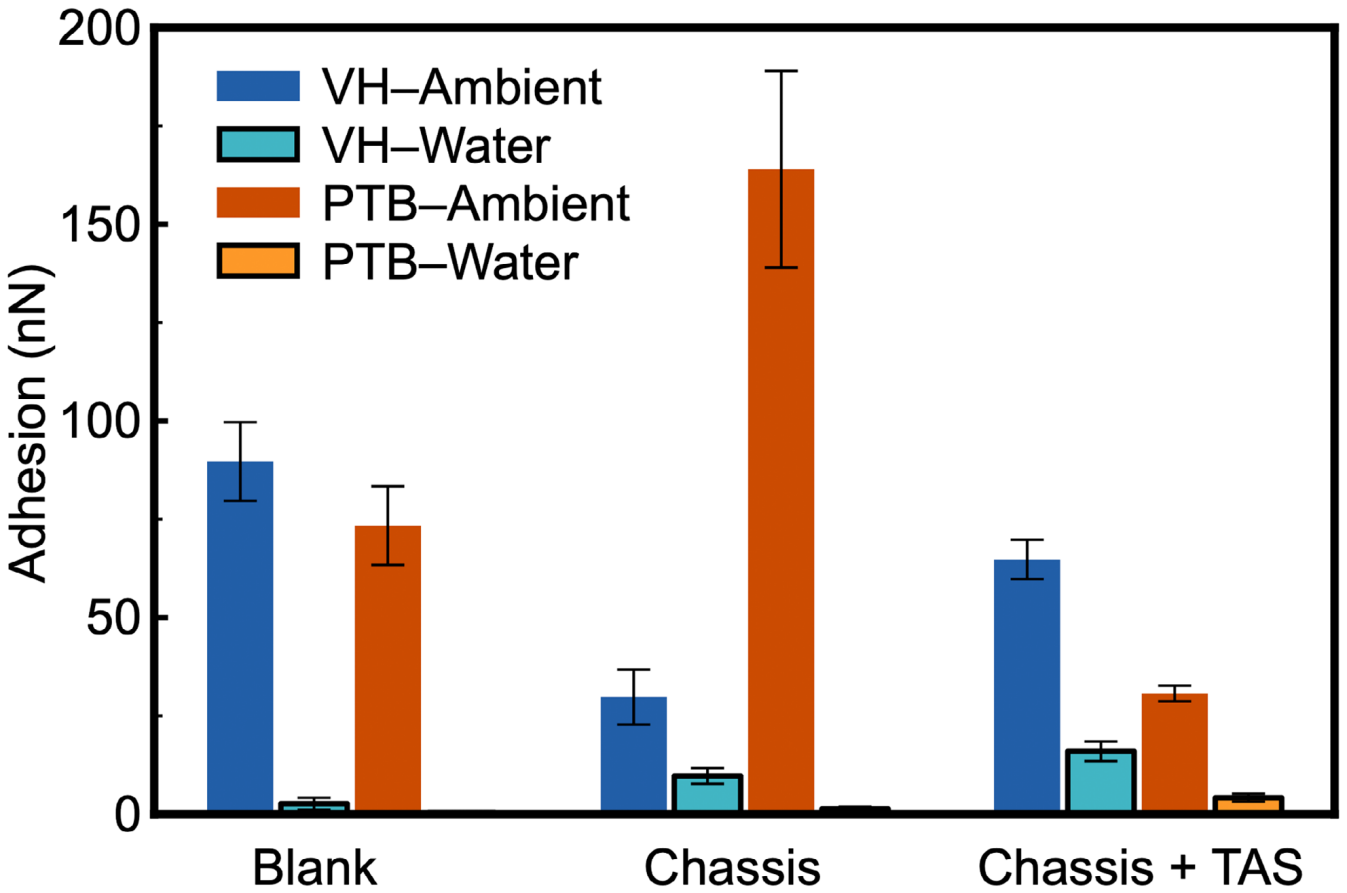
Adhesion force between −COOH‐coated AFM cantilever and hair surface before treatment (virgin hair, platinum‐bleached hair) and after treatment (none, chassis alone and chassis + TAS) in ambient air and in de‐ionized water.

Adhesion force on both VH and PTB in ambient increases nearly 10 times than that acquired by using −CH_3_ functionalized tips (Figure 5). Such substantial enhancement is primarily due to the improved capillary force that was introduced by the hydrophilic cantilever. It is also probable that electrostatic force plays a role in the localized aqueous environment. The capillary force introduced by the hydrophilic cantilever is so great in ambient that it could overshadow the effect introduced by the difference in hydrophobicity between VH and PTB, and the adhesion was found to be slightly greater on VH than that on PTB. This is also confirmed by instability of the baseline of force curves due to an excessive attraction between cantilever and hair surface. Strong capillary effect was also observed on the hair samples treated with chassis. The ratio between VH and PTB (VH_adhesion_ / PTB_adhesion_) that were treated by chassis/TAS increased from 1.5 to 2.1, which can potentially be used to assess the presence of amino groups as they contribute primarily towards the electrostatic interactions on the PTB surface.

For measurements in water, the overall adhesion was reduced substantially when the capillary force was depleted. However, the −COOH cantilever introduces an electrostatic interaction that could facilitate attractive interaction with cationic surfactant or polymers that treatments (chassis or chassis/TAS) deposit on to the surface. This underpins the increased adhesion observed for both sets of samples (VH and PTB) in Figure 5.

The approaching part of the force curve acquired (Figure S4) displays a long‐range repulsion between the cantilever and PTB surface, starting from approximately 100 nm separation, which is characteristic for electrostatic interaction. Although the charge distribution and coverage are not known, this is very likely due to a negatively charged surface, which is consistent with our surface free energy measurements that suggest PTB is much more polar than VH (Table 2). Therefore, the substantial reduction in adhesion for untreated VH and PTB is explained by repulsive electrostatic interaction between PTB and −COOH cantilever. Upon the deposition of chassis, the surface chemistry of VH and PTB shows a drastic difference: VH becomes positively charged, whilst PTB remains negatively charged. The typical elongation pattern that was observed by −CH_3_ cantilever is also visible here on hair treated with silicone. The difference between VH and PTB shows that the deposited chassis/TAS film is loosely organized on VH. In light of those results, there are several factors for consideration in the building up of the gel network or gel network/TAS on hair based on the physical and chemical properties of the surface.

**TABLE 2:** Surface energy data obtained from contact angles on virgin hair and platinum‐bleached hair using hexadecane for the non‐polar liquid and water for the polar liquid.

Hair and location in 4 g‐8″ tress	Surface free energy (mJ m^−2^)	
	Non‐polar component	Polar component
Virgin hair (middle of tress)	25.7 ± 2.6	0.3 ± 0.3
Platinum‐bleached hair (middle of tress)	27.5 ± 1.5	42.1 ± 1.7

The results so far suggest the following:

1. Functional groups present on hair surface control deposition amount and kinetics, driven by the attractive electrostatic interaction. This is supported by the variation between VH and PTB against −COOH cantilever.
2. Upon treatment by various formulations, functional groups exposed to the environment are dependent on the chemical nature and molecular architecture of the actives used.
3. Once hair fibres are exposed to a full formulation, there is a competitive adsorption between different actives, which controls the molecular configuration and arrangement within the deposited film. For example, adhesion on VH is always greater than that on PBT, which is likely due to the configuration of cationic species in the deposited film. A possible deposition mechanism is that the gel network adsorbed on the VH surface via hydrophobic interaction, and hence exposing the cationic groups towards the liquid environment, whilst it is driven by electrostatic attraction to the PTB surface, and hence showing no attractive interaction with the negatively charged AFM cantilever, as illustrated in Figure 3.

### Hair surface adhesion against hydroxyl (−OH) functional group

To further evaluate the impact of surface chemistry on the overall adhesion, hydroxyl (−OH) terminated cantilevers were used in addition to −CH_3_ and −COOH functional groups investigated. It is equally hydrophilic, but is less polar than −COOH group, and less readily to interact with amine groups of TAS.

**FIGURE 6:**
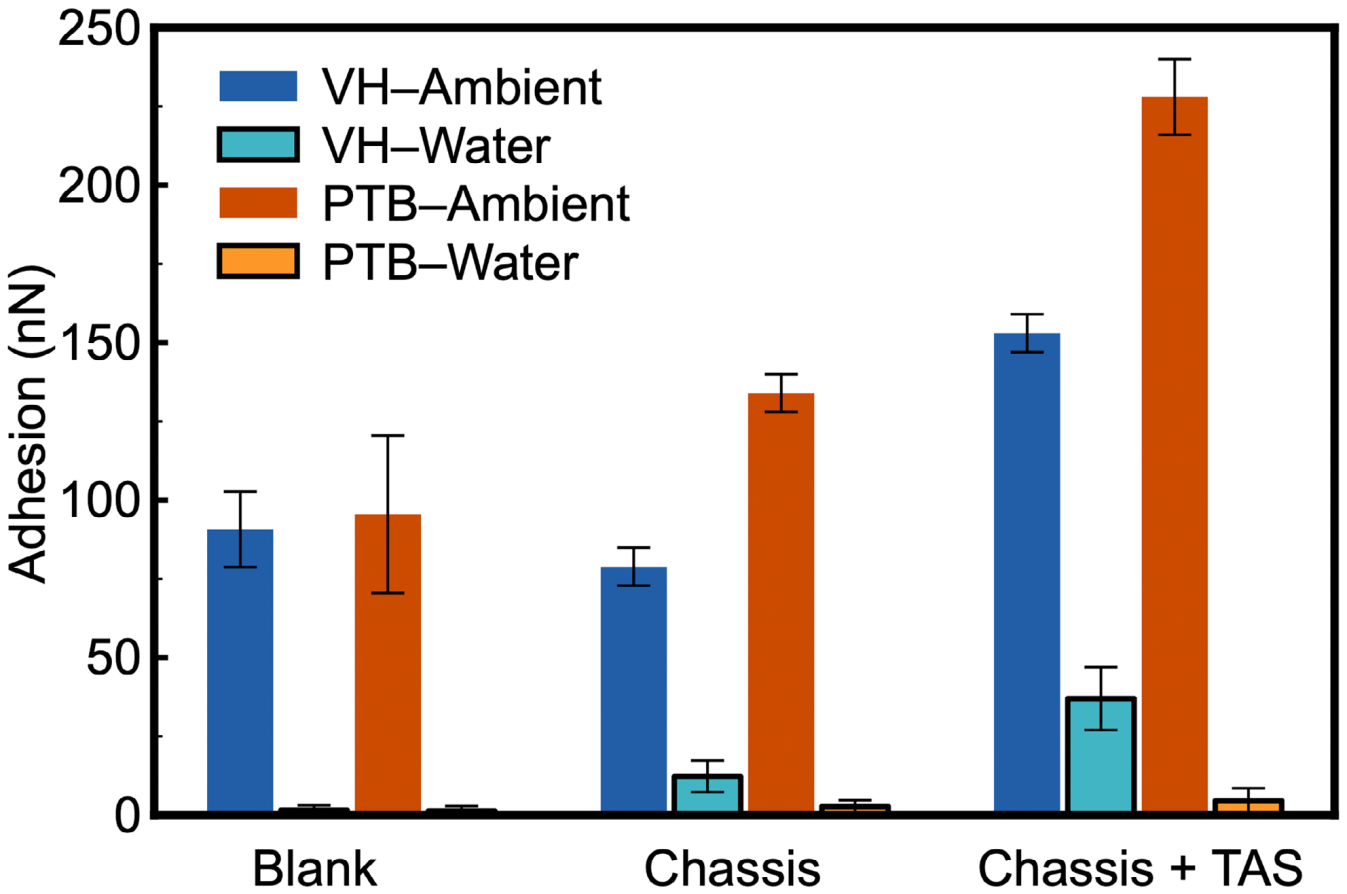
Adhesion force between −OH‐coated AFM cantilever and hair surface before treatment (virgin hair, platinum‐bleached hair) and after treatment (none, chassis alone and chassis/TAS) in ambient air and in de‐ionized water.

The results acquired in ambient (Figure 6) show a similar trend to what we observed for both −CH_3_ and −COOH cantilevers because capillary force or viscous force plays the most significant role in determining surface adhesion in ambient. It is determined by the hydrophobicity/hydrophilicity of the hair fibre and AFM cantilever rather than the charge density of the AFM cantilever. For blank fibres, including both VH and PTB, the adhesion values are very close, which could be interpreted as no significant difference between the middle regions of VH and PTB sampled in the present work. Upon treatment of chassis and chassis/TAS, adhesion increases and is greater for PTB than for VH. There is clearly a strong attraction between the hair and the cantilever, which overshadows the stretching events observed on TAS in the other conditions. Once again, this supports the hypothesis that hair surface adhesion in ambient is controlled by capillary force, which is determined by the functional groups available on the surfaces, as well the surrounding environment.

Once the hair samples were immersed in water, the strong adhesion observed in ambient no longer exists, due to the absence of capillary force (Figure 6). It is worth noting that the electrostatic repulsion is not as strong as that induced by −COOH surface functionalization. Unlike the adhesion measured by −CH_3_ and −COOH functionalized cantilevers, which are driven by hydrophobic interaction and electrostatic interaction, adhesion to bare hair substrates is similar for both VH and PTB when −OH cantilever was used. This suggests that the chemistry at the interface has a critical role in improving the deposition of future hair care products. It also indicates that the conformation of deposited molecules, including both gel network and TAS, is a critical factor for consideration, in addition to the fundamental knowledge generated by the chemical analysis. Adhesion of −OH cantilevers on VH and PTB treated with chassis or chassis/TAS displayed a similar trend to that by −COOH. This is highly likely due to the similar chemical nature between them.

Representative force curves in water (Figure S6) indicate there was a clear repulsion when the probe was approaching the PBT hair substrate due to the electrostatic interaction. The saw‐tooth pattern has been repeatedly observed with the VH sample treated with chassis/TAS. It is indisputable that the polymer chains are being stretched during the retraction process of the cantilever, which suggests that silicone molecules adapt a different configuration to those deposited on the PTB.

### Hair surface adhesion against amine (−NH_2_ ) functional group

Lastly, positively charged cantilevers, using −NH_2_ functionalized self‐assembled monolayer, were deployed to probe the hair surface interaction. This, in principle, would not only affect the capillary force due to the magnitude of surface free energy, but have a significant influence on the electrostatic interaction when measurements taking place in water.

**FIGURE 7:**
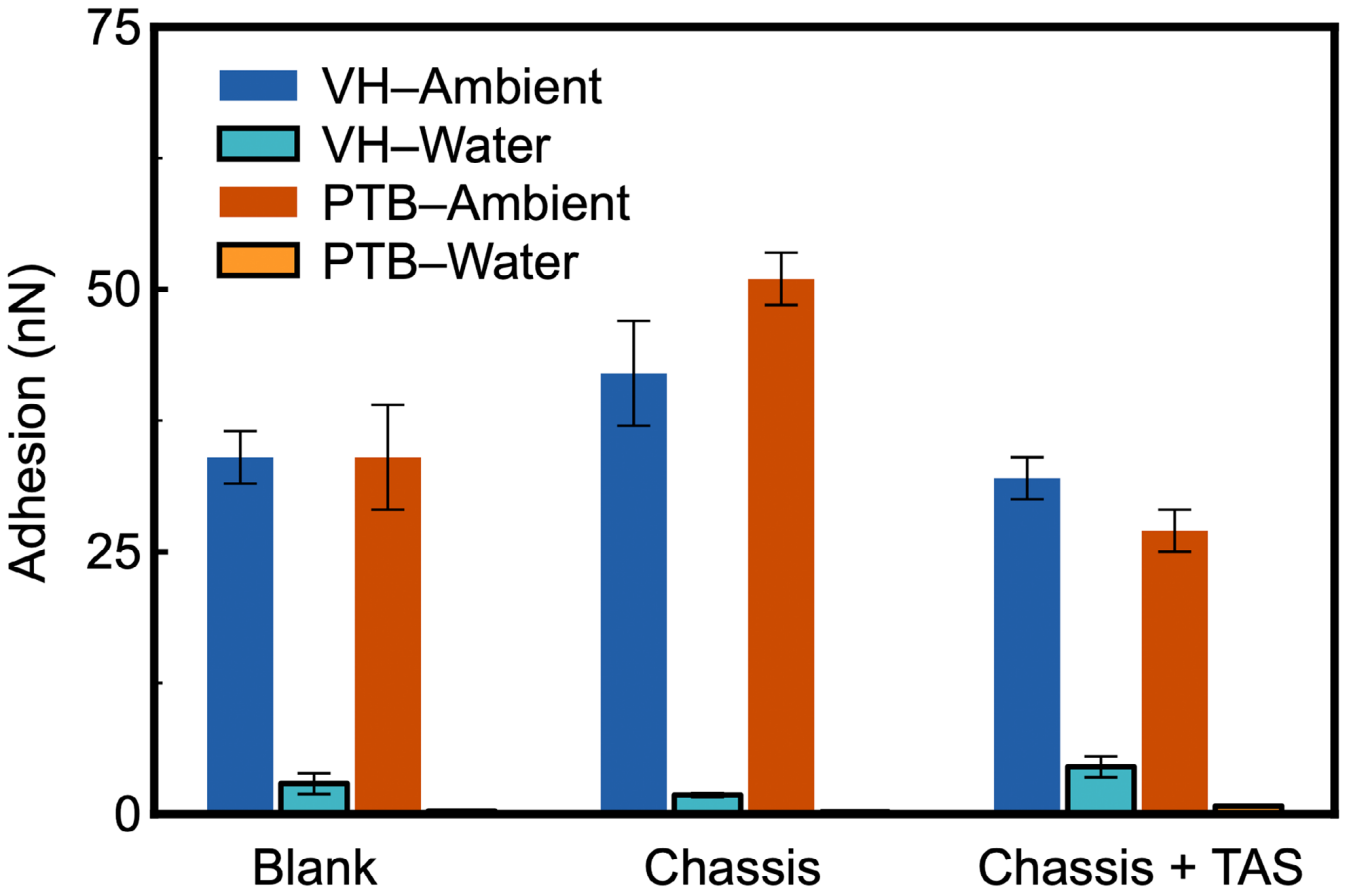
Adhesion force between –NH_2_‐coated AFM cantilever and hair surface before treatment (virgin hair, platinum‐bleached hair) and after treatment (none, chassis alone and chassis + TAS) in ambient air and in de‐ionized water.

Averaged adhesion data generated by the −NH_2_ functionalized cantilevers are presented in Figure 7. In ambient, the charge and charge density of the cantilever do not have a significant role whilst surface hydrophilicity is much more critical. Adhesion on bare substrates (~34 nN) is not as significant as that produced by −COOH or −OH (~90 nN). However, it is much greater than that measured by −CH_3_ (~9 nN). This is because −COOH group is more polar (hydrophilic) than −NH_2_ [33]. The similar values of adhesion on VH and PTB are consistent with the results in ambient, generated by the other types of surface chemistry.

**FIGURE 8:**
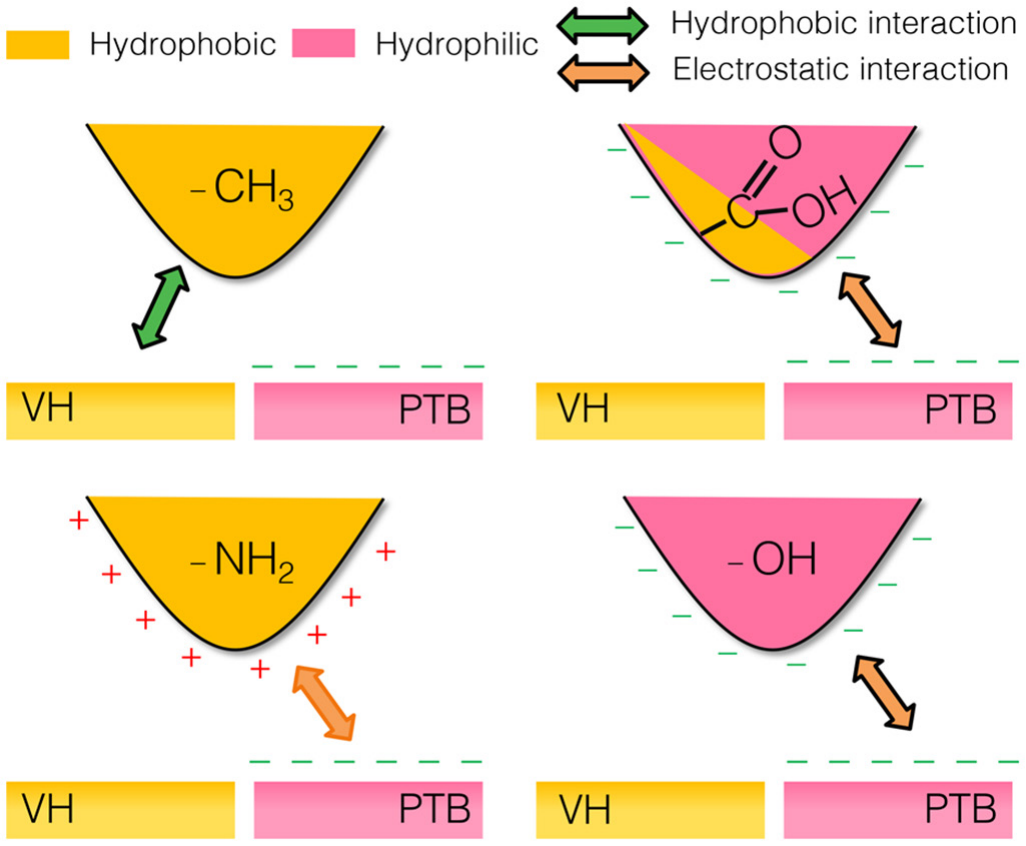
Schematic diagrams illustrating the driving forces of interaction between the functionalized AFM probes and the blank surface of VH and PTB hair in water.

As seen with the other probes, the adhesion of –NH_2_ functionalized cantilever is stronger on PTB treated with chassis than on VH with the same treatment. However, the variation between VH and PTB is not as substantial as that measured by −COOH, which is likely due to the reduced hydrophilicity of −NH_2_. Once again, result acquired on substrates treated with chassis/TAS shows that there is a slightly greater adhesion to the VH. This observation agrees with what is observed upon exposure to water even if the adhesion force is reduced greatly compared to that in ambient. It can be seen from the corresponding force curves in Figure S8 that the pulling events show an extraordinary characteristic that is commonly associated with loosely arranged polymer layer. This was observed with polymer brush, as well extra‐cellular polymeric substances [32, 37]. It confirms our hypothesis that the deposited film is much more swollen in water and much less compact and loosely organized on VH than on PTB, and prefers to interact with the amine AFM tip. On blank hair, it is surprising that the adhesion against PTB is much lower than that on VH because this does not agree with what we commonly believe that damaged hair is more polar [3, 14, 23], hence the cationic functional groups integrated in both surfactants and silicones to enhance the deposition. There are however other possibilities for consideration, such as the presence of polar but not negatively charged groups on the PTB surface (Figure 8).

## CONCLUSION

This work highlights the great potential of using AFM to measure surface interactions on hair fibre, which enables understanding of deposition and feel properties of conditioning actives on different hair types. The method was demonstrated successfully to quantify differences in surface characteristics, particularly surface adhesion, between virgin and highly bleached hair, with or without treatment of conditioning chassis alone and the chassis with aminosilicone. The capability of functionalizing AFM cantilevers purposely to probe a particular molecular interaction on hair surface was presented with high sensitivity. It was feasible to establish the effects of chemistry and conformation of chemical groups on hair, upon carefully prepared chemistry on AFM cantilever and the environment in which the surface interaction is probed. We show that the deposition of gel network and silicones is driven by different forces, depending on the hair type. Our data, in particularly the adhesion result generated by the −CH_3_ functionalized cantilever shows that hydrophobic interaction is a key driver for deposition on virgin hair, but electrostatic interactions could be the most important on bleached hair. Conformation of chassis components (polymer network, silicones) is also very different between virgin and bleached hair, which is determined by the underlying hair surface properties. The adhesion data between a bare hair fibre (virgin, platinum bleached, or any other) and an AFM cantilever functionalized by different chemistries could be used to identify the immobilization mechanism of surface actives used in hair conditioning products for such particular type of hair. The study also highlights the possibility of a distribution of polar but not necessarily negatively charged groups on the damaged hair.

Future development needs to be focused on damaged hair where hydrophobic adhesion is a much less contributor to deposition mechanisms. Fundamental understanding of the chemical groups available on the substrates (e.g. bleached hair) could be further explored using different probes to identify the role of surface groups such as SO_4_^2−^ and Ca^2+^. It was noticed that location dependent properties of bleached hair show a different trend than that of virgin hair. This may open to a more detailed study on model substrates of different cuticle arrangements to understand the role of cuticle loss on surface adhesion.

## Supporting information


Appendix S1.
