# Supplementary material for: Hair surface interactions against different chemical functional groups as a function of environment and hair condition
Source: Int J Cosmet Sci. 2023 Mar 14;45(2):224–35. doi: 10.1111/ics.12834 (PMC10946710; doi:10.1111/ics.12834)
Supplement: Supplementary file 1 — Appendix S1. [file ICS-45-224-s001.docx]

SUPPORTING INFORMATION

Hair surface interactions against different chemical functional groups as a function of environment and hair condition

AFM FORCE CURVES

## Methyl (-CH3) terminated tip

| 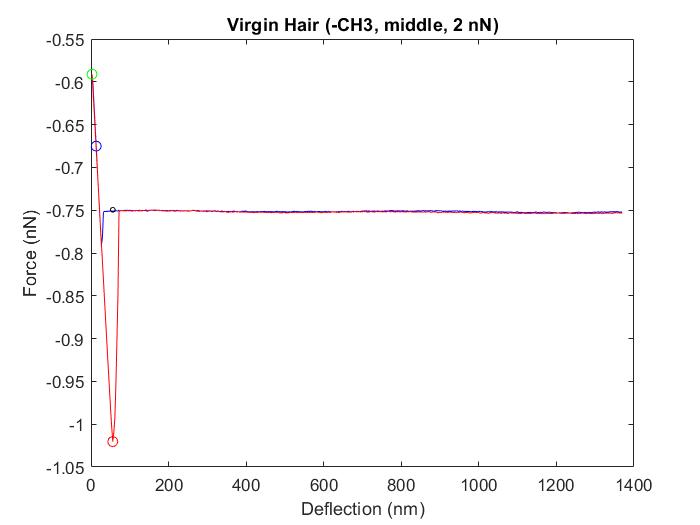 | 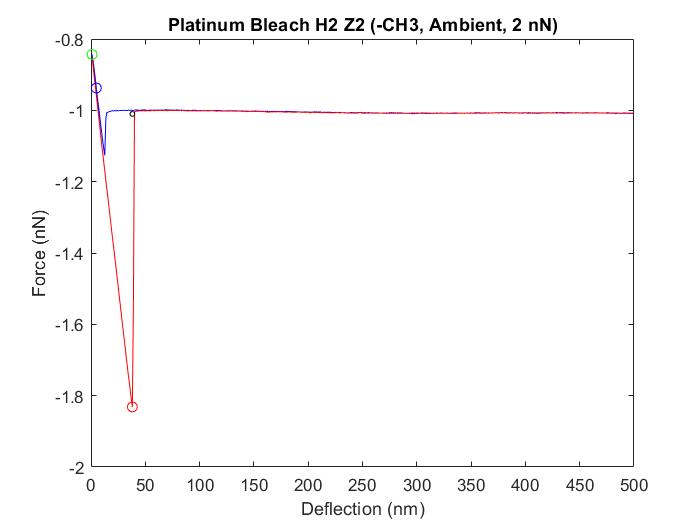 |
| --- | --- |
|  |  |
| 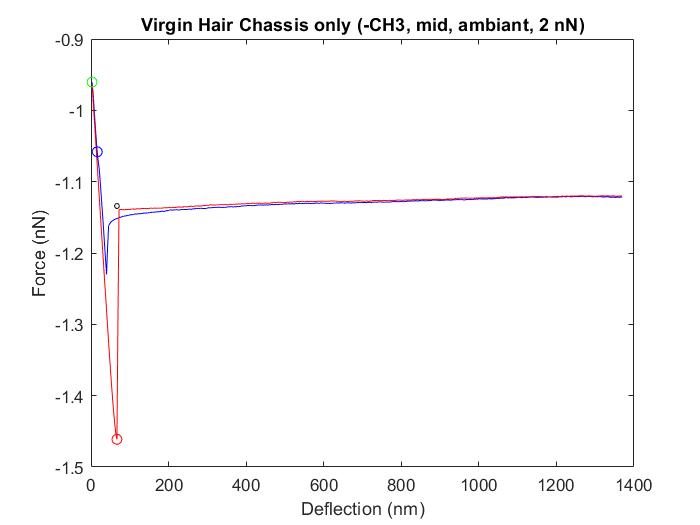 | 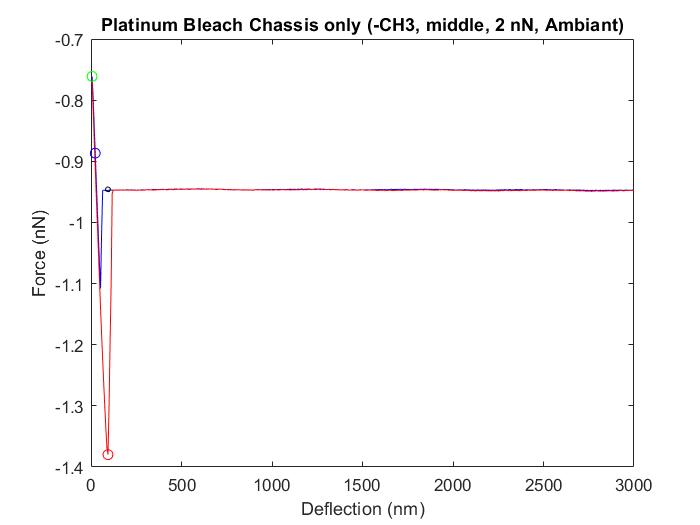 |
|  |  |
| 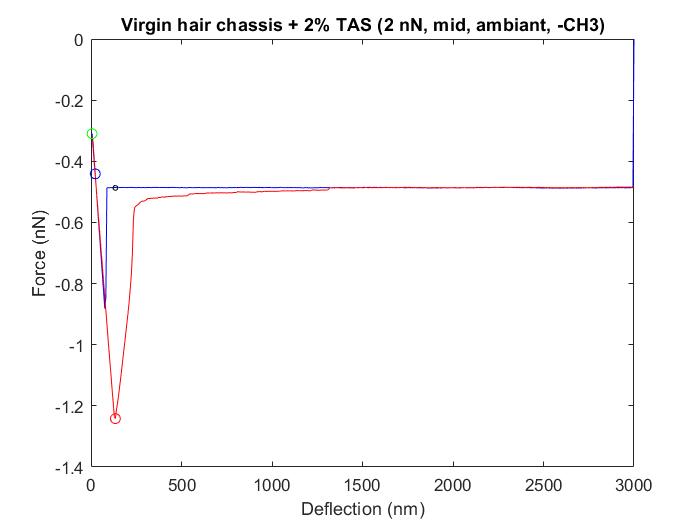 | 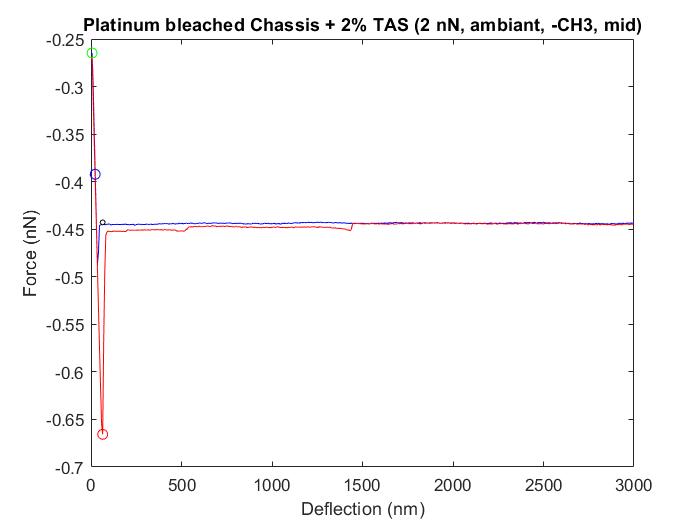 |
|  |  |

Figure S1. Representative force curves for -CH_3_ in ambient for the 6 hair samples: a) Virgin Hair (VH), b) Platinum Bleached Hair (PTB), c) VH Chassis only, d) PTB Chassis only, e) VH Chassis + 2% TAS, f) PTB Chassis + 2% TAS.

| 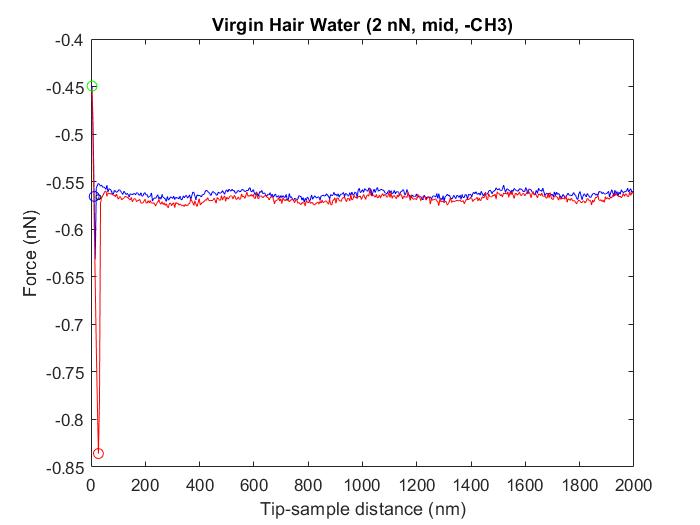 | 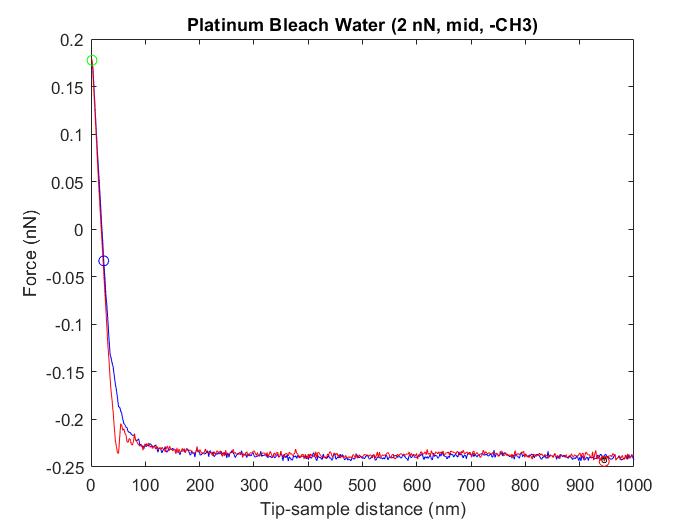 |
| --- | --- |
|  |  |
| 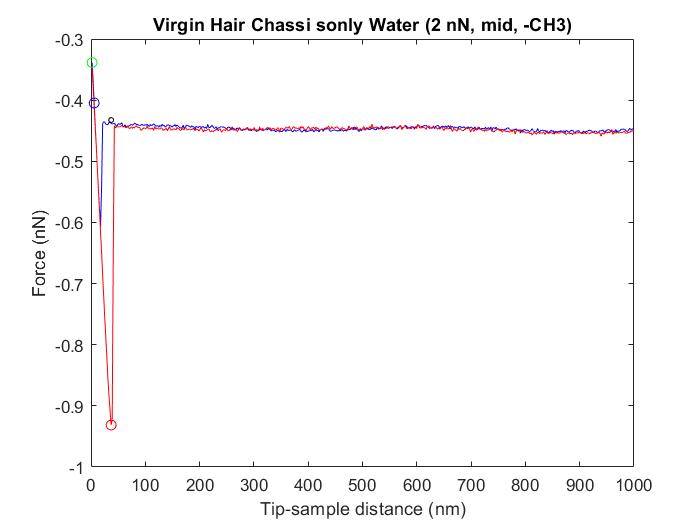 | 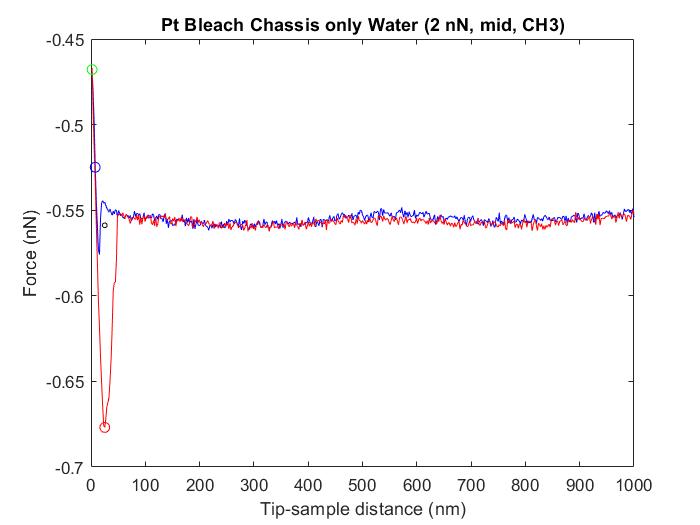 |
|  |  |
| 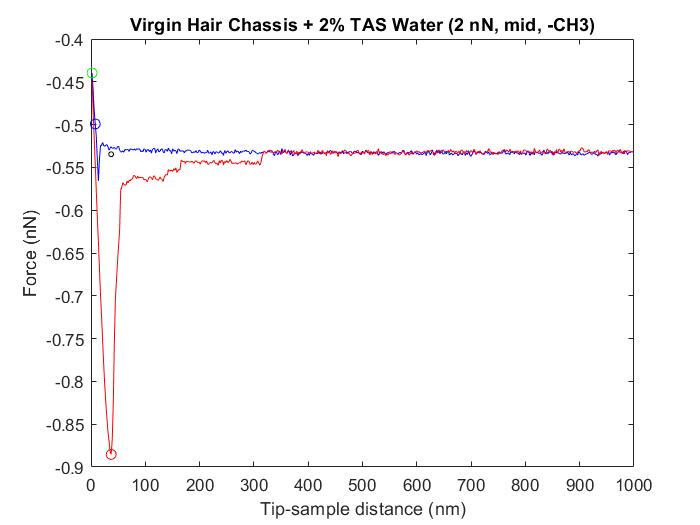 | 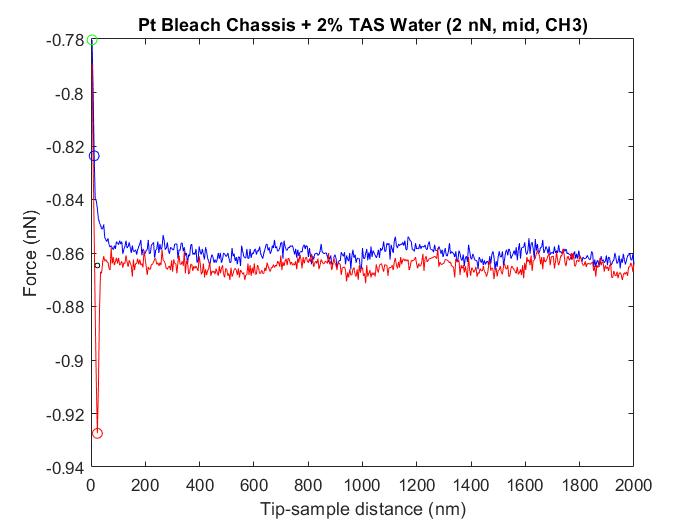 |
|  |  |

Figure S2. Representative force curves for -CH_3_ in water for the 6 hair samples: a) Virgin Hair (VH), b) Platinum Bleached Hair (PTB), c) VH Chassis only, d) PTB Chassis only, e) VH Chassis + 2% TAS, f) PTB Chassis + 2% TAS.

Table S1. Adhesion values corresponding to the force curves for -CH_3_ in ambient and in water for the 6 hair samples: a) Virgin Hair (VH), b) Platinum Bleached Hair (PTB), c) VH Chassis only, d) PTB Chassis only, e) VH Chassis + 2% TAS, f) PTB Chassis + 2% TAS

| CH_3_ probe | VH in ambient | | VH in water | | PTB in ambient | | PTB in water | |
| --- | --- | --- | --- | --- | --- | --- | --- | --- |
|  | Average | STD | Average | STD | Average | STD | Average | STD |
| Blank | 8.9 | 0.5 | 8.3 | 1.25 | 9.9 | 1 | 0.2 | 0.5 |
| Chassis | 16.1 | 3 | 5.9 | 1 | 16.9 | 3 | 1.7 | 0.75 |
| Chassis + 2% TAS | 29.8 | 3 | 1.9 | 0.5 | 19.8 | 3 | 1.6 | 1 |

Carboxylic acid (-COOH) terminated tip

| 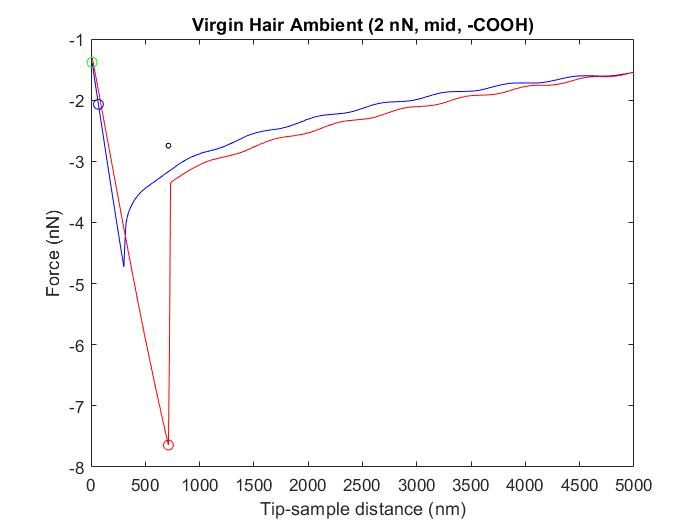 | 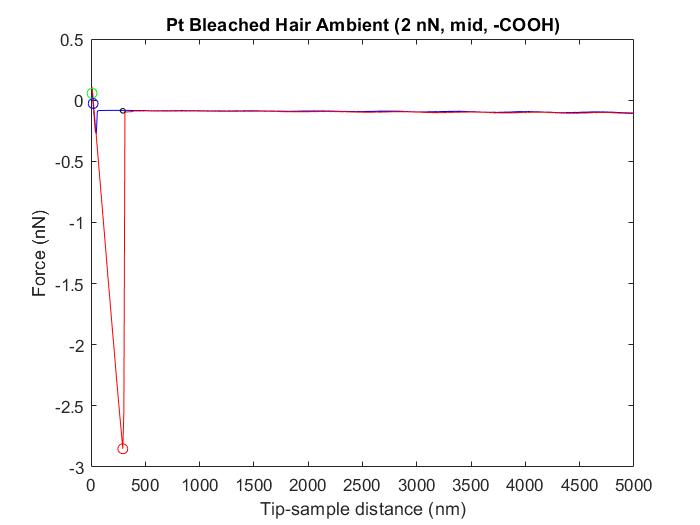 |
| --- | --- |
|  |  |
| 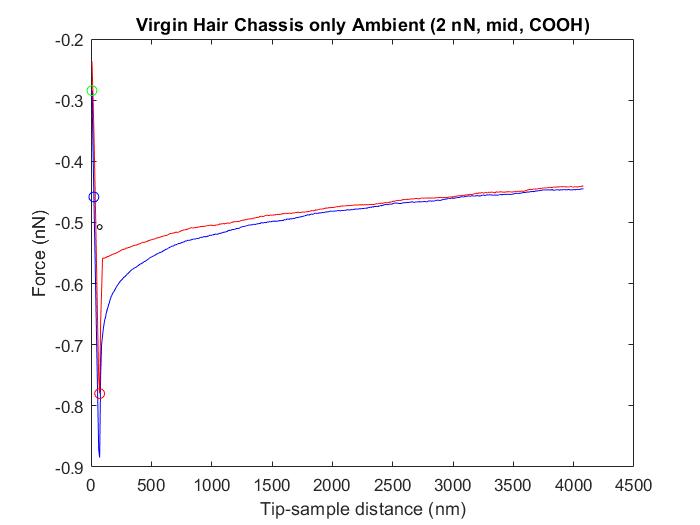 | 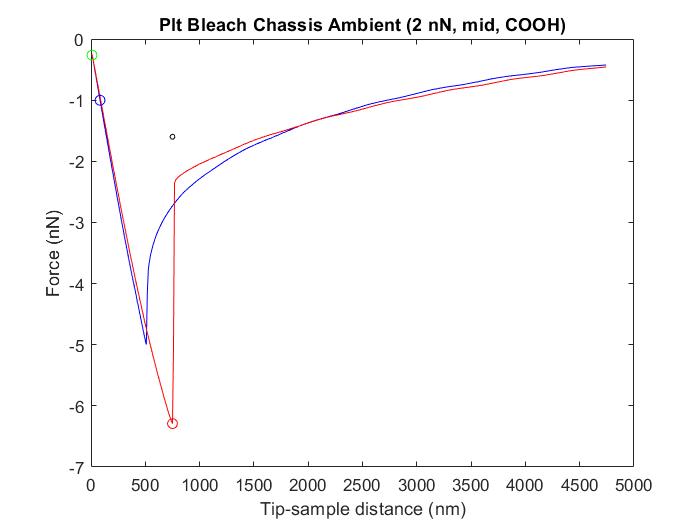 |
|  |  |
| 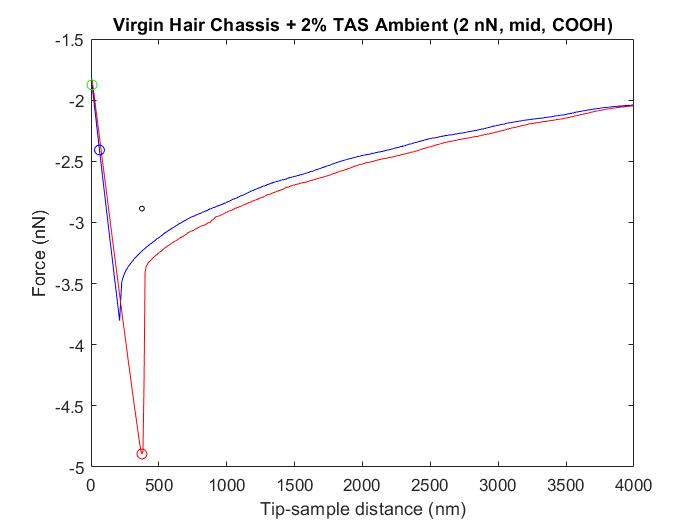 | 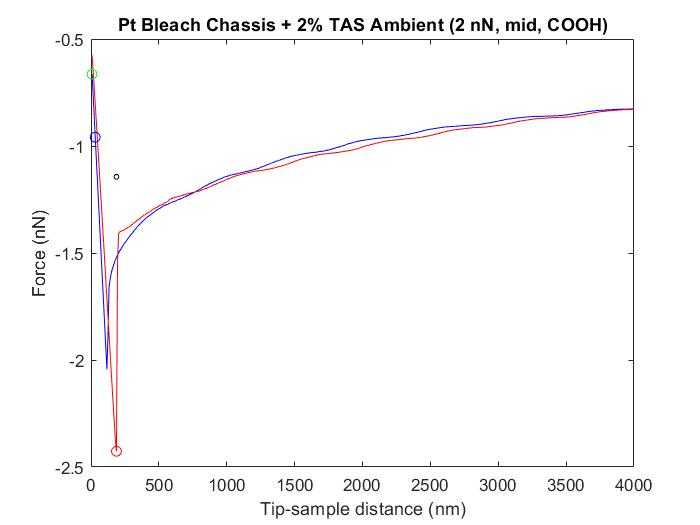 |
|  |  |

Figure S3. Representative force curves for COOH in ambient for the 6 hair samples: a) Virgin Hair (VH), b) Platinum Bleached Hair (PTB), c) VH Chassis only, d) PTB Chassis only, e) VH Chassis + 2% TAS, f) PTB Chassis + 2% TAS.

| 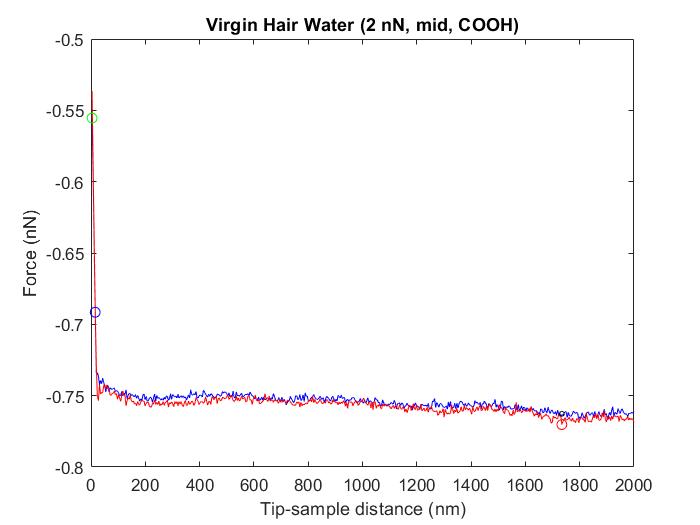 | 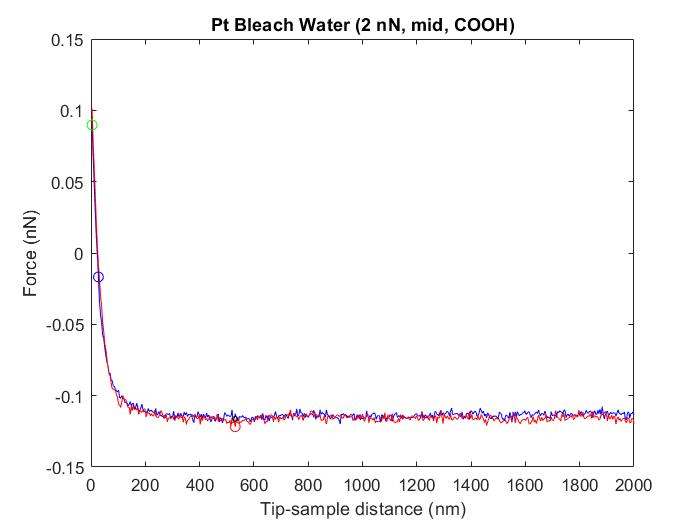 |
| --- | --- |
|  |  |
| 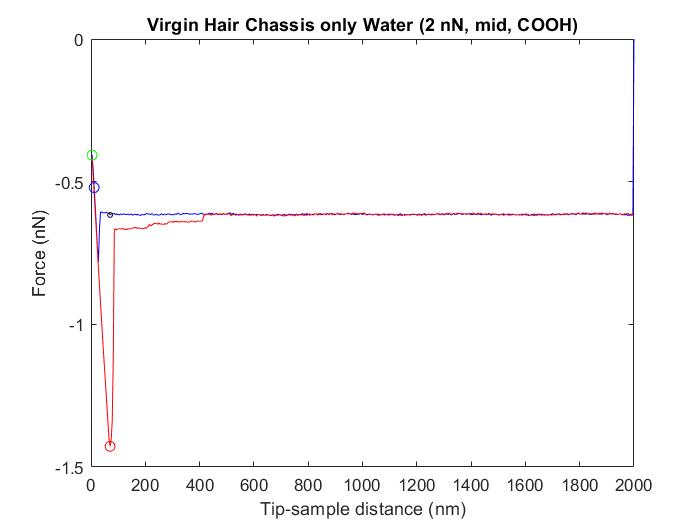 | 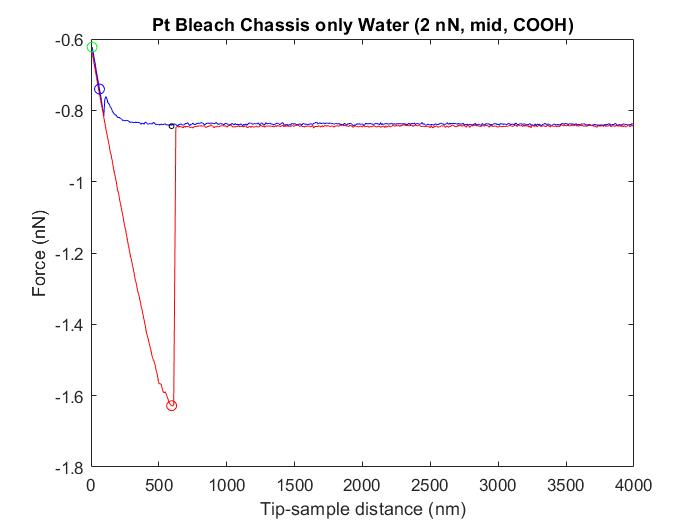 |
|  |  |
| 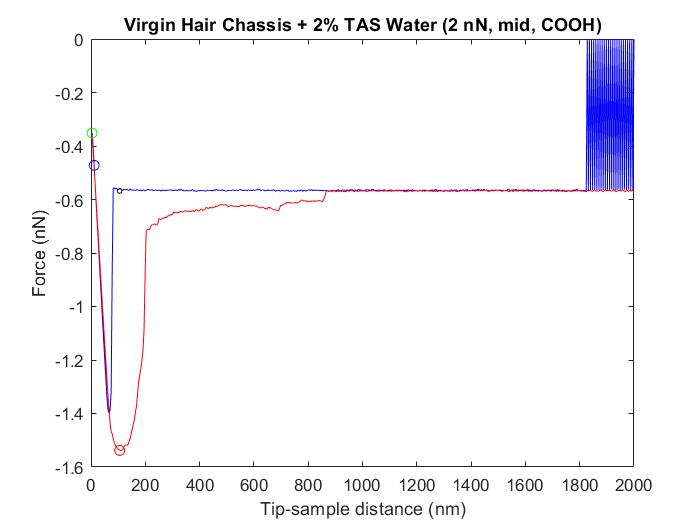 | 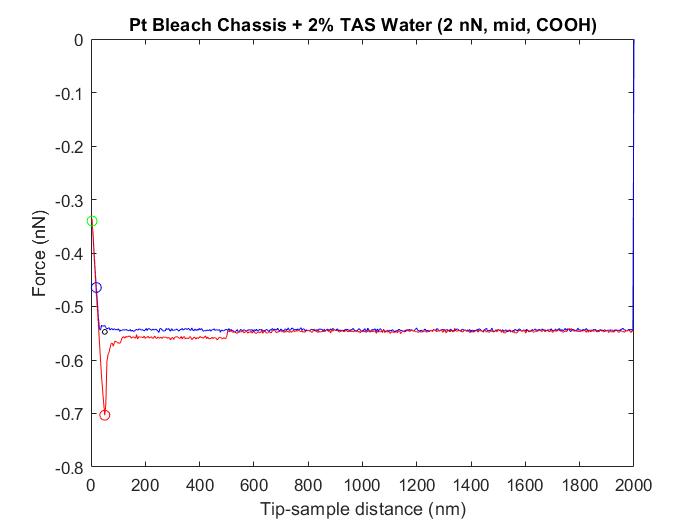 |
|  |  |

Figure S4. Representative force curves for -COOH in water for the 6 hair samples: a) Virgin Hair (VH), b) Platinum Bleached Hair (PTB), c) VH Chassis only, d) PTB Chassis only, e) VH Chassis + 2% TAS, f) PTB Chassis + 2% TAS.

Table S2. Adhesion values corresponding to the force curves for -COOH in ambient and in water for the 6 hair samples: a) Virgin Hair (VH), b) Platinum Bleached Hair (PTB), c) VH Chassis only, d) PTB Chassis only, e) VH Chassis + 2% TAS, f) PTB Chassis + 2% TAS

| COOH probe | VH in ambient | | VH in water | | PTB in ambient | | PTB in water | |
| --- | --- | --- | --- | --- | --- | --- | --- | --- |
|  | Average | STD | Average | STD | Average | STD | Average | STD |
| Blank | 89.7 | 10 | 2.68 | 1.5 | 73.4 | 10 | 0.413 | 0.1 |
| Chassis | 29.8 | 7 | 9.74 | 2 | 164 | 25 | 1.37 | 0.5 |
| Chassis + 2% TAS | 64.8 | 5 | 16 | 2.5 | 30.7 | 2 | 4.18 | 1 |

Hydroxyl (-OH) terminated tip

| 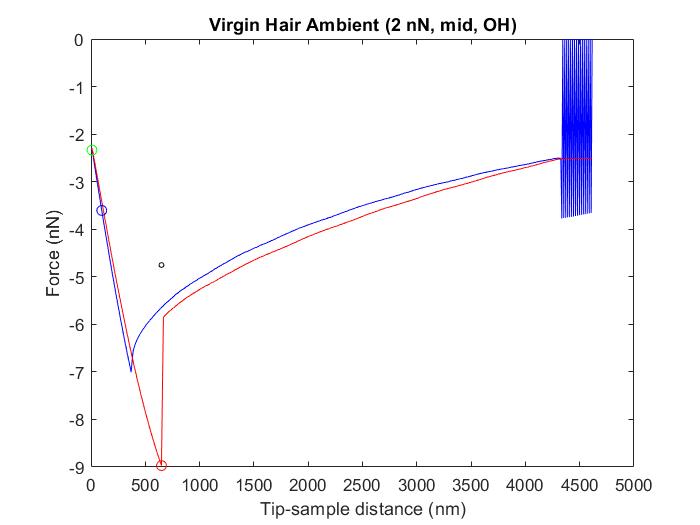 | 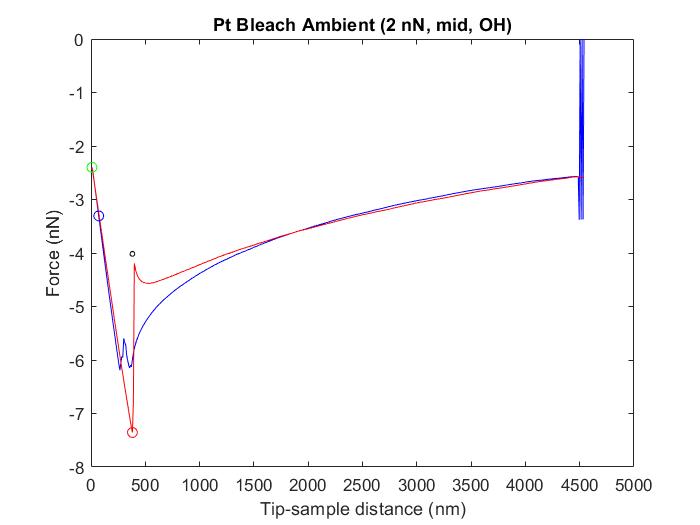 |
| --- | --- |
|  |  |
| 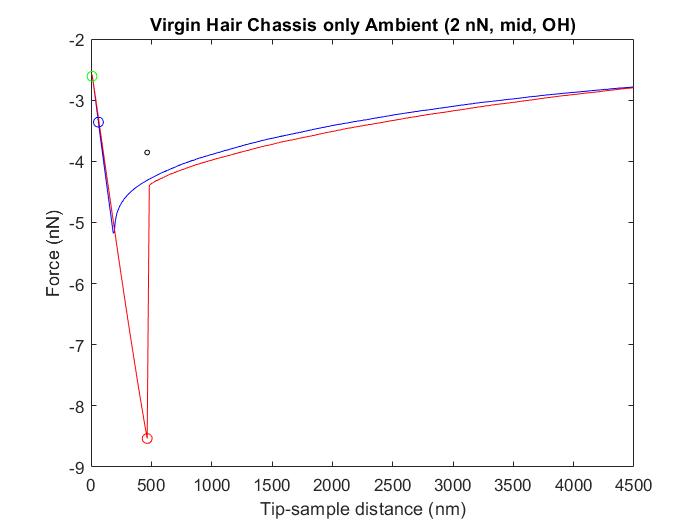 | 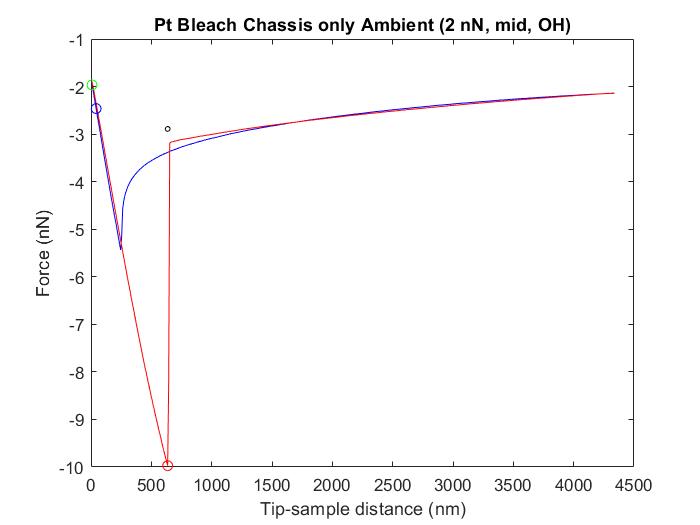 |
|  |  |
| 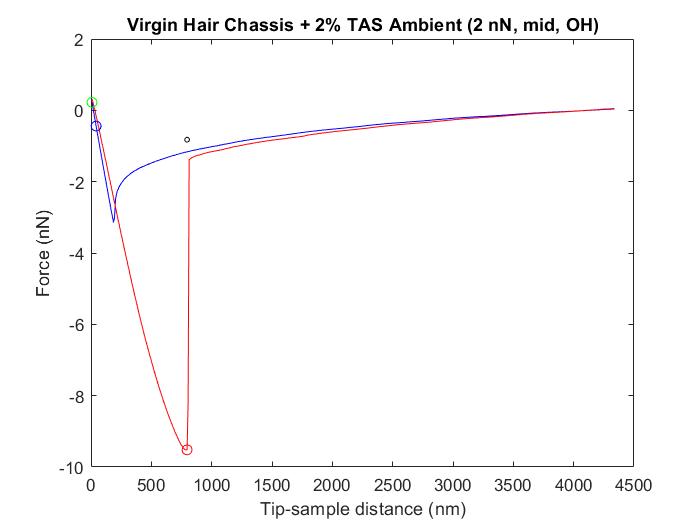 | 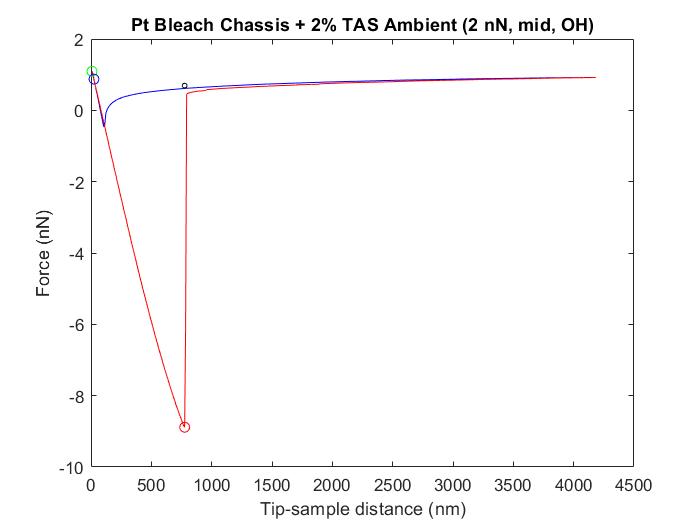 |
|  |  |

Figure S5. Representative force curves for -OH in ambient for the 6 hair samples: a) Virgin Hair (VH), b) Platinum Bleached Hair (PTB), c) VH Chassis only, d) PTB Chassis only, e) VH Chassis + 2% TAS, f) PTB Chassis + 2% TAS.

| 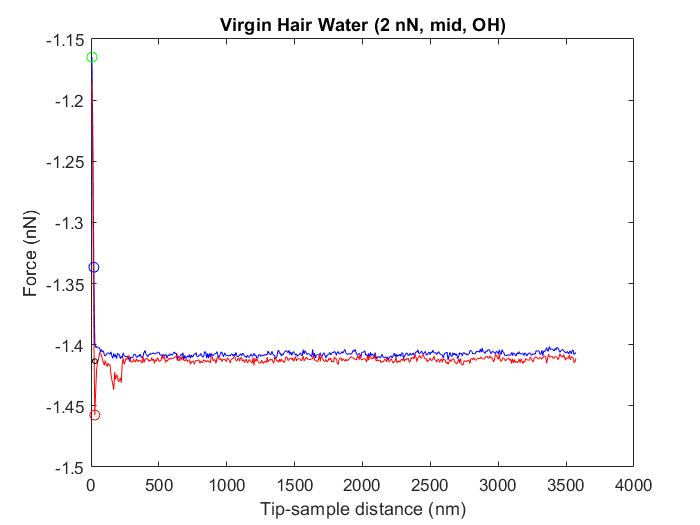 | 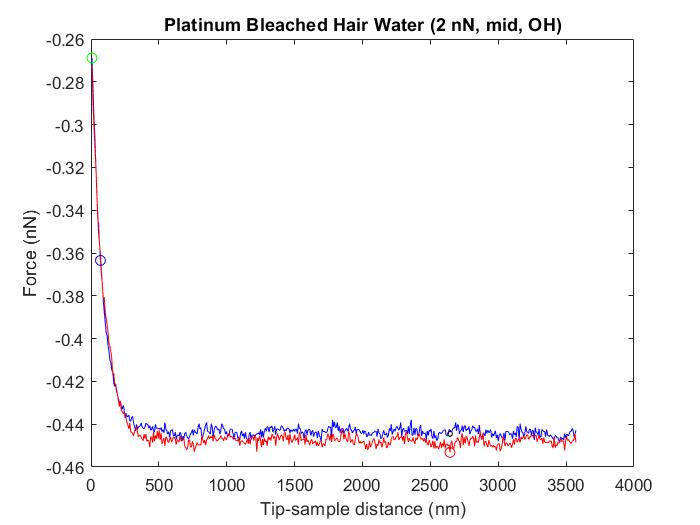 |
| --- | --- |
|  |  |
| 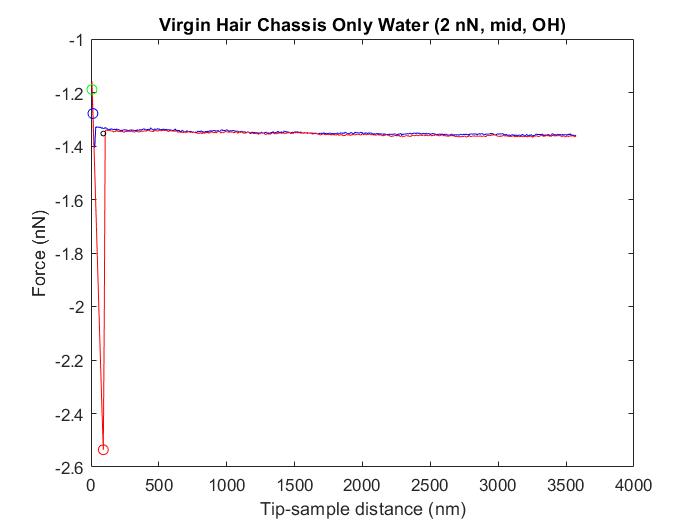 | 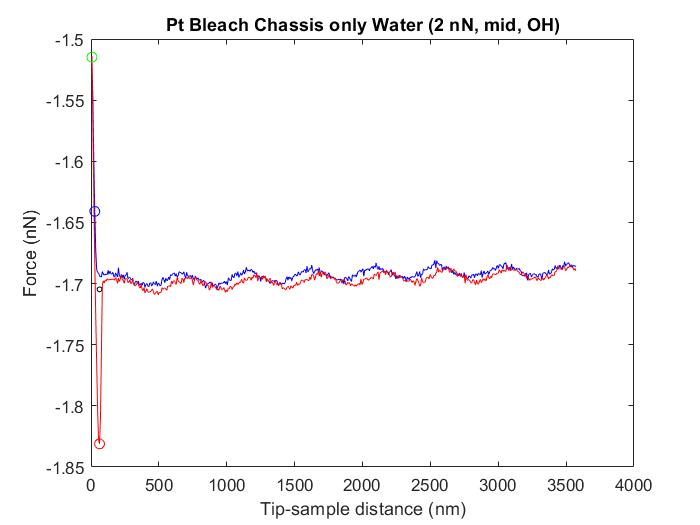 |
|  |  |
| 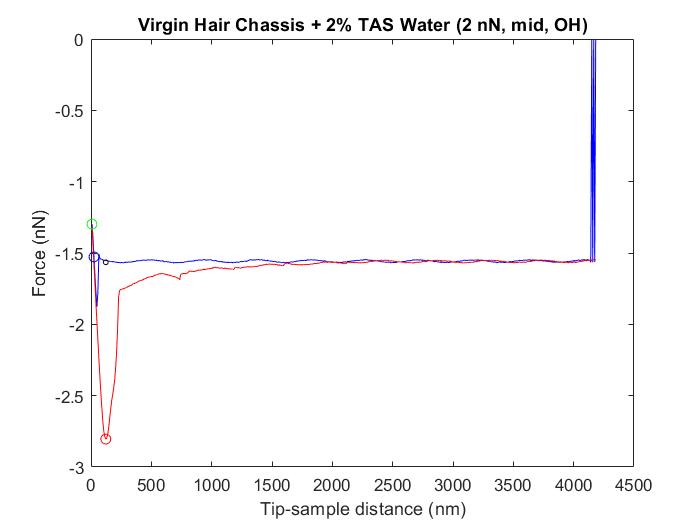 | 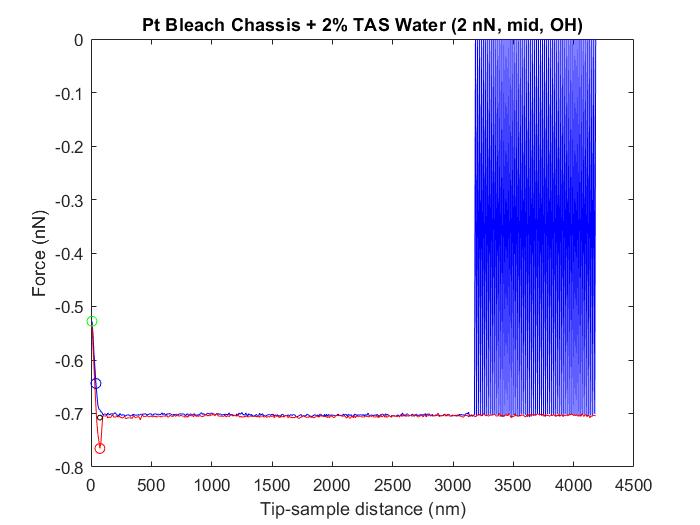 |
|  |  |

Figure S6. Representative force curves for -OH in water for the 6 hair samples: a) Virgin Hair (VH), b) Platinum Bleached Hair (PTB), c) VH Chassis only, d) PTB Chassis only, e) VH Chassis + 2% TAS, f) PTB Chassis + 2% TAS.

Table S3. Adhesion values corresponding to the force curves for -OH in ambient and in water for the 6 hair samples: a) Virgin Hair (VH), b) Platinum Bleached Hair (PTB), c) VH Chassis only, d) PTB Chassis only, e) VH Chassis + 2% TAS, f) PTB Chassis + 2% TAS

| OH probe | VH in ambient | | VH in water | | PTB in ambient | | PTB in water | |
| --- | --- | --- | --- | --- | --- | --- | --- | --- |
|  | Average | STD | Average | STD | Average | STD | Average | STD |
| Blank | 90.7 | 12 | 1.6 | 1.5 | 95.5 | 25 | 1.37 | 1.5 |
| Chassis | 78.9 | 6 | 12.3 | 5 | 134 | 6 | 2.71 | 2 |
| Chassis + 2% TAS | 153 | 6 | 37 | 10 | 228 | 12 | 4.58 | 4 |

Amine (-NH_2_) terminated tip

| 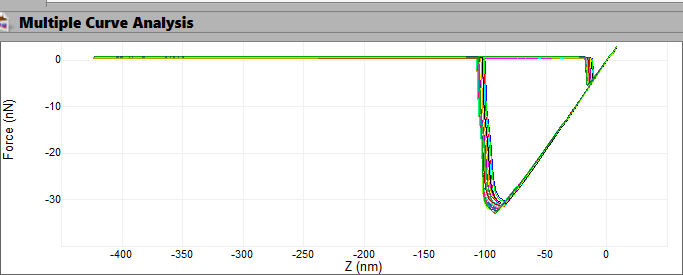 | 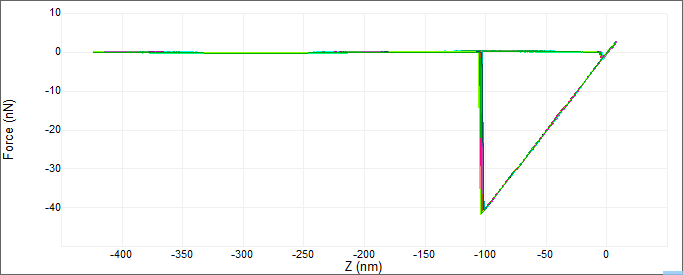 |
| --- | --- |
|  |  |
| 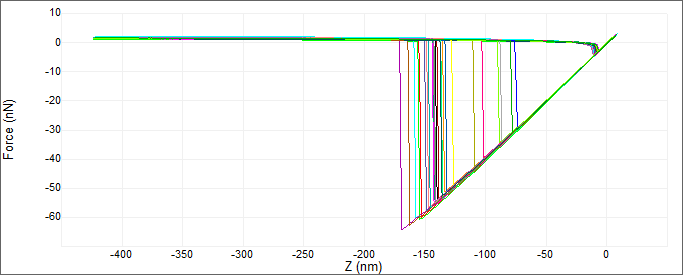 | 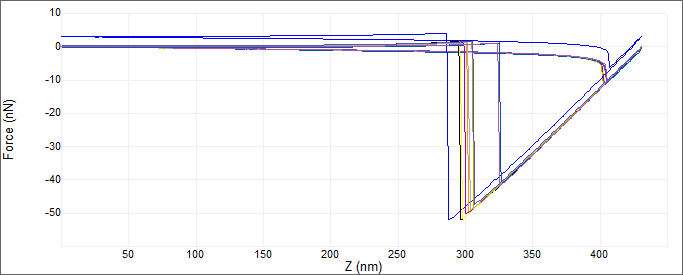 |
|  |  |
| 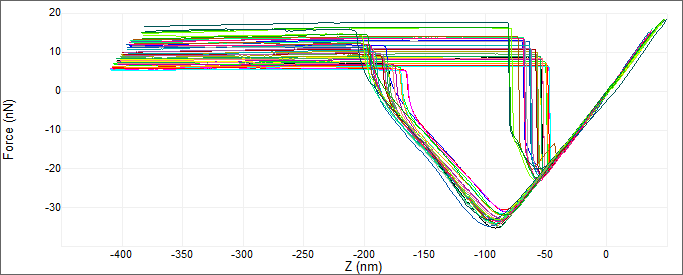 | 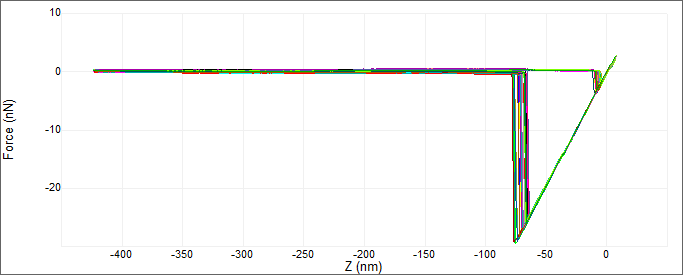 |
|  |  |

Figure S7: Representative force curves for NH_2_ in ambient for the 6 hair samples: a) Virgin Hair (VH), b) Platinum Bleached Hair (PTB), c) VH Chassis only, d) PTB Chassis only, e) VH Chassis + 2% TAS, f) PTB Chassis + 2% TAS.

| 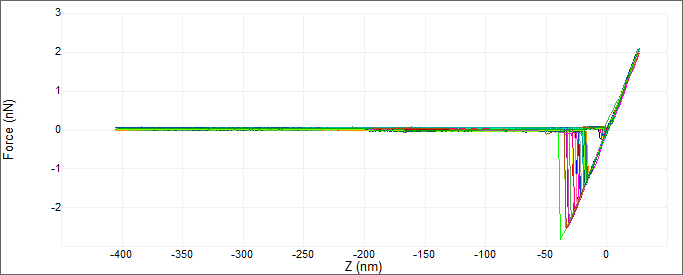 | 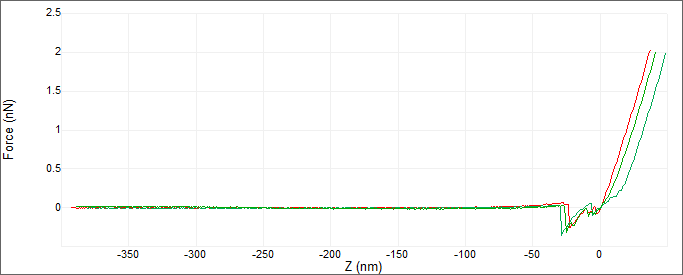 |
| --- | --- |
|  |  |
| 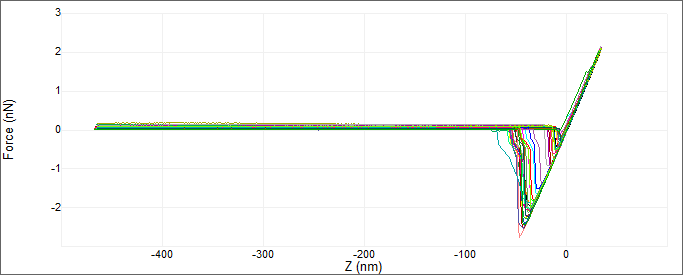 | 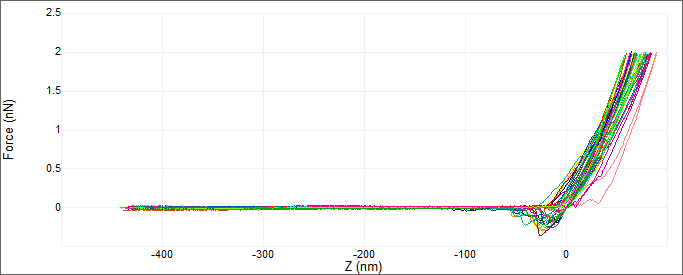 |
|  |  |
| 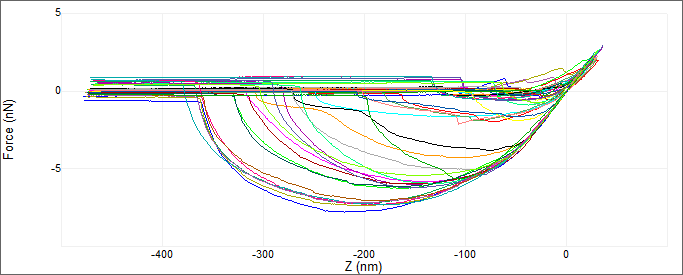 | 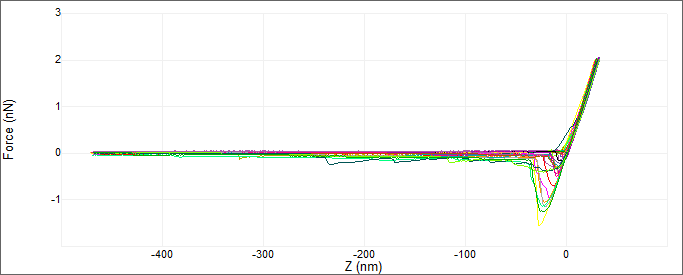 |
|  |  |

Figure S8. Representative force curves for -NH_2_ in water for the 6 hair samples: a) Virgin Hair (VH), b) Platinum Bleached Hair (PTB), c) VH Chassis only, d) PTB Chassis only, e) VH Chassis + 2% TAS, f) PTB Chassis + 2% TAS.

Table S4. Adhesion values corresponding to the force curves for -NH_2_ in ambient and in water for the 6 hair samples: a) Virgin Hair (VH), b) Platinum Bleached Hair (PTB), c) VH Chassis only, d) PTB Chassis only, e) VH Chassis + 2% TAS, f) PTB Chassis + 2% TAS

| NH_2_ probe | VH in ambient | | VH in water | | PTB in ambient | | PTB in water | |
| --- | --- | --- | --- | --- | --- | --- | --- | --- |
|  | Average | STD | Average | STD | Average | STD | Average | STD |
| Blank | 34 | 2.5 | 2.9 | 1 | 34 | 5 | 0.31 | 0.05 |
| Chassis | 42 | 5 | 1.8 | 0.2 | 51 | 2.5 | 0.21 | 0.05 |
| Chassis + 2% TAS | 32 | 2 | 4.5 | 1 | 27 | 2 | 0.76 | 0.05 |
